# Immune cell regulatory networks in chronic obstructive pulmonary disease: mechanistic analysis from innate to adaptive immunity

**DOI:** 10.3389/fimmu.2025.1651808

**Published:** 2025-10-16

**Authors:** Hui Li, Yingqi Wang, Hongxia Duan, Yidie Bao, Xinliao Deng, Yucheng He, Qian Gao, Peijun Li, Xiaodan Liu

**Affiliations:** ^1^ Shanghai University of Traditional Chinese Medicine, School of Rehabilitation Medicine, Shanghai, China; ^2^ The Second Rehabilitation Hospital of Shanghai, Shanghai, China; ^3^ Engineering Research Center of Intelligent Rehabilitation of Traditional Chinese Medicine, Shanghai University of Traditional Chinese Medicine, Shanghai, China; ^4^ Shanghai Sports University (SSU), Shanghai, China; ^5^ School of Sports and Health Sciences, Shanghai University of Sport, Rehabilitation Institute of Shanghai University of Traditional Chinese Medicine, Shanghai, China

**Keywords:** COPD, immune system, innate immunity, autoadaptive immunity, immune cell regulatory networks

## Abstract

**Background:**

Chronic Obstructive Pulmonary Disease (COPD) is a leading cause of global mortality, characterized by chronic inflammation and abnormal immune responses in the lower airways. Recent studies have highlighted the critical role of immune function in the pathogenesis and progression of COPD. The disease is characterized by abnormal immune responses in the lower respiratory tract, with its progression associated with the infiltration of innate and adaptive inflammatory immune cells into the lungs and the formation of lymphoid follicles, mediated by cytokines and inflammasomes. Increasing evidence suggests that cell-mediated immunity has an important role in the pathogenesis of COPD, which is characterized by immune senescence leading to decreased resistance to infection, enhanced neutrophil and macrophage activation, T-cell infiltration, and aberrant B-cell activity, all of which combine to contribute to airway inflammation and lung injury in patients with COPD.

**Objective:**

This review aimed to explore the pivotal role of the immune system in COPD and its therapeutic potential.

**Methods:**

We reviewed, categorized, and summarized literature on immunity and COPD published in the last five years from Web of Science and PubMed databases.

**Results:**

This study elucidates the pivotal role of immune dysregulation in COPD pathogenesis, particularly the dysfunctional transition from innate to adaptive immunity. We delineate how specific immune cell populations—including macrophages, neutrophils, and T-lymphocytes—contribute to sustained airway inflammation and lung injury in COPD through aberrant activation, infiltration, and impaired function. Mechanistically, key features of this dysregulation involve aberrant cytokine signaling pathways and defective resolution of inflammation. These insights reveal potential therapeutic targets for immunomodulatory strategies aimed at interrupting the chronic inflammatory cascade, restoring immune homeostasis, and mitigating infection susceptibility in COPD. Promising approaches highlighted include targeting specific cytokines, modulating macrophage polarization states, and enhancing mucosal immune defenses.

## 1 Introduction

Chronic Obstructive Pulmonary Disease (COPD) represents a prevalent respiratory disorder characterized by persistent airflow limitation, manifesting clinically as progressive worsening of dyspnea, chronic cough, sputum production, and ultimately predisposing to life-threatening complications including respiratory and cardiac failure when left uncontrolled. The disease course typically alternates between stable phases and acute exacerbations (AECOPD), with the latter significantly accelerating pulmonary function decline. Current epidemiological projections indicate COPD will emerge as the third leading global cause of mortality by 2030 (1), constituting a substantial public health challenge due to its escalating prevalence, debilitating morbidity, and substantial socioeconomic burden.

**Figure 1:**
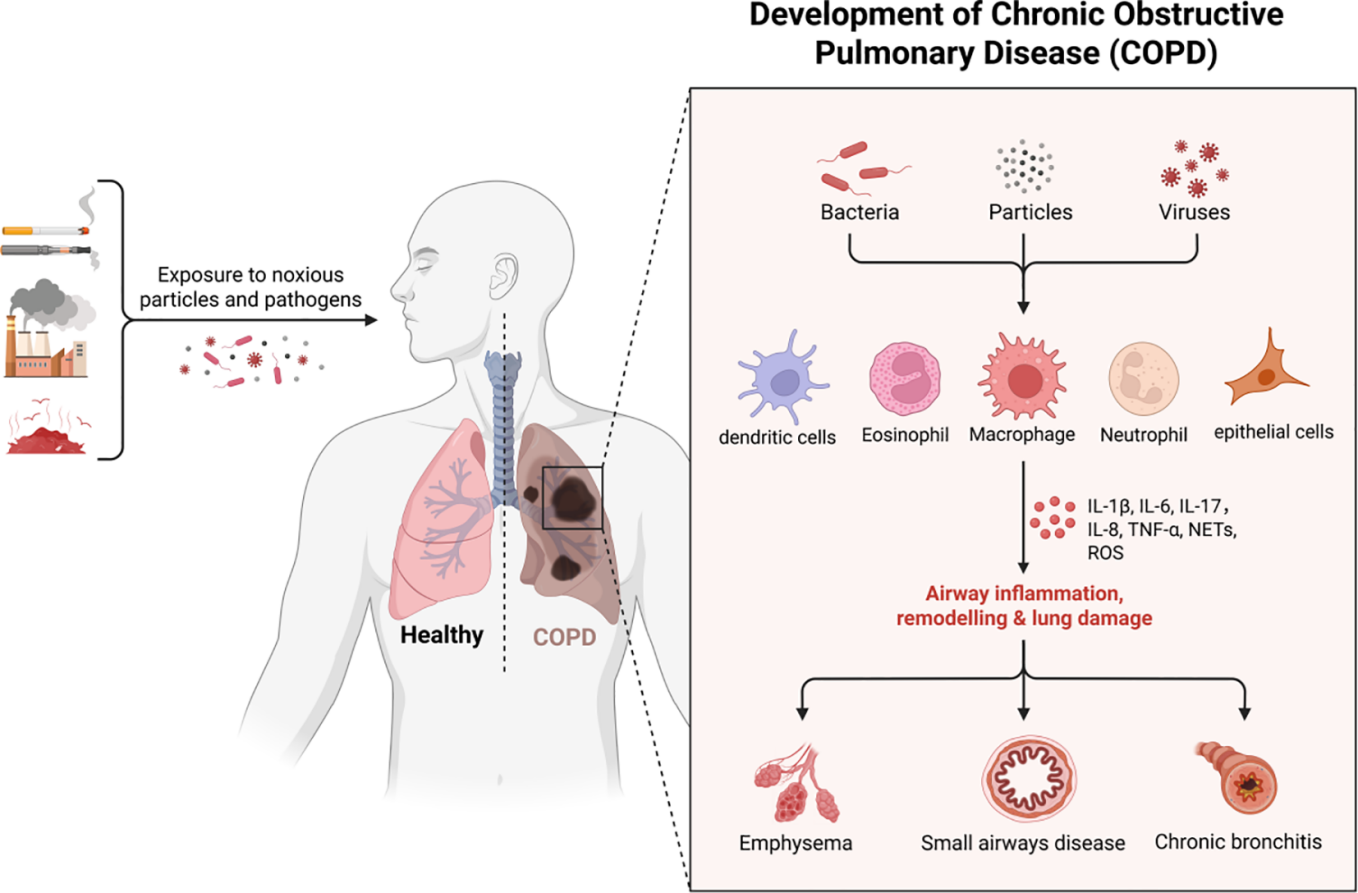
The pathogenesis of COPD.

The human immune system orchestrates a sophisticated defense network against pathogenic invasion through complementary innate and adaptive mechanisms. Innate immunity provides immediate, non-specific protection via physical barriers (e.g., epithelial linings), phagocytic cells (neutrophils, macrophages), and humoral components (complement proteins, antimicrobial peptides) (2). In contrast, adaptive immunity mediates antigen-specific responses through lymphocyte-mediated memory formation and targeted effector functions. While these subsystems employ distinct cellular players (dendritic cells, T/B lymphocytes) and molecular mediators (cytokines, antibodies), they synergistically integrate through both humoral (complement, antibodies) and cell-mediated (phagocytosis, cytotoxic killing) mechanisms (**Figure 1**). Notably, specialized neurovascular barriers (blood-brain, blood-cerebrospinal fluid) compartmentalize central nervous system immunity from peripheral immune activity (3).

Pathogen evolution drives continuous immune adaptation through conserved defense strategies across phylogeny. Prokaryotic organisms employ restriction enzymes as primitive antiviral defenses, while ancestral eukaryotes developed foundational mechanisms including phagocytosis, defensin production, and complement-like systems preserved in modern plants and invertebrates. Jawed vertebrates evolved sophisticated adaptive immunity featuring somatic recombination of antigen receptors, enabling immunological memory that enhances secondary responses (4). This evolutionary arms race has shaped a multi-layered defense paradigm: 1) Immediate innate detection via pattern recognition receptors (PRRs) 2) Coordinated inflammatory recruitment 3) Antigen-specific adaptive targeting 4) Long-term immunological memory - all critical for maintaining host-pathogen equilibrium. This study categorized and summarized literature related to immunity and COPD published in the Web of Science and PubMed databases over the past five years (**Tables 1**, **2**).

**Table 1:** Articles related to immunization and COPD.

Number	Mechanism	Results	Year
1 (5)	Emphysema/COPD	PD-1, FGFR1, PIK3CA, PTEN和p16	2019
2 (6)	Neutrophile granulocyte	An important component of the innate immune system	2024
3 (7)	The phagosomes of macrophages versus apoptotic cells	Rubicon reduce,LAP lose balance	2021
4 (8)	Bar cells	Rod cells in the airways of COPD patients	2023
5 (9)	Epithelial cells, and dendritic cells	Therapeutic potential of immunomodulation during COPD progression	2020
6 (10)	Extracellular matrix	Immune activity-mediated	2022
7 (11)	Enteric microorganisms	Gut microbes and the immune system	2022
8 (12)	Macrophage	Long type (lfTSLP) and shorter TSLP subtype (sfTSLP)	2024
9 (13)	Macrophages, ILC 2, and DCs	Significantly improved chronic lung disease	2023
10 (14)	Cytokine environment and T cell nicotinic acetylcholine receptor (nAChR), macrophages	The ratio of Th 1 and Th 2 cells in peripheral blood	2023
11 (15)	Neutrophil extracellular trap (NET)	It promotes AEC proliferation, nuclear factor κ B (NF- κ B) activation, NF- κ B, dependent cytokine and type I interferon production, and DC maturation	2024
12 (16)	Immunosenescence	Immunosenescence increases the low susceptibility to COPD	2019
13 (17)	Intestinal lung axis	The APC (macrophages, dendritic cells, B cells) have membrane receptors called the pattern recognition receptor (PR)	2022
14 (18)	Cell ageing	Activation of the cell cycle regulators p21 (CIP1), p16 (INK 4, and the cell tumor antigen p53	2024
15 (19)	Lung-intestinal crosstalk	Both the lung and the intestine belong to the common mucosal immune system (CMIS)	2023
16 (20)	Innate immune cells	The innate lymphocyte (ILC) family plays an important role in COPD	2024
17 (21)	Activation of the macrophages	IFN- γ can activate macrophages and initiate control of pathogens	2021
18 (22)	Novel chemokines	Association between DIO 2, chemokines, and COPD	2019
19 (23)	Hypergrade blood eosinophils	Relationship between blood eosinophil count, clinical characteristics, and bronchial gene expression in COPD patients	2020
20 (24)	Neutrophile granulocyte	Neutrophils-mediating the deterioration of COPD	2020
21 (25)	Respiratory viral infection	Multiple immunomodulators and inflammatory signaling pathways of the host-viral response	2020
22 (26)	RNA(lncRNA)	On immune-related MHC I, antigen presentation, and adaptive immune responses.	2022
23 (27)	Airway epithelial dysfunction	Mitochondrial damage mediates the pro-cellular senescence effects induced by mitochondrial reactive oxygen species	2024
24 (28)	Macrophage	COPD lung macrophages have less ability to undergo apoptotic cells	2020
25 (29)	Lung iron excess	Iron sequestration may lead to immune cell dysfunction, leading to an increased frequency of infection	2022
26 (30)	RNA immune regulation of COPD	N6-methyladenosine (m6A) methylation regulates gene expression and function by affecting post-transcriptional RNA modifications	2023
27 (31)	RNA	Bioinformatics analysis to identify key genes and underlying molecular mechanisms in COPD	2019
28 (32)	microRNAs (miRNA)	Potential miRNA biomarkers for the early diagnosis and therapeutic targets of COPD	2019
29 (33)	Particulate matter and cell phagocytosis	Fine particulate matter (PM2.5) affects phagocytosis in macrophages in COPD mice through the actin-associated protein (Arp) 2/3 complex	2019
30 (34)	Inducible bronchial-associated lymphoid tissue (iBALT)	iBALT Altering the biological outcome of the disease	2020
31 (35)	Pro-inflammatory and anti-inflammatory immune responses	The progression of inflammation mediated by the immune response underwent a temporal analysis that focused on the balance between Th 17 and Treg responses.	2019
32 (36)	Airway epithelial cells	The role of the major Ca ^(2)^ signaling pathways found in airway epithelial cells and their contribution to airway inflammation, mucosal ciliary clearance, and surfactant production.	2024
33 (37)	Dysbiosis of the gut and lung microbiota	The T-helper-cell (Th) 17/regulatory T-cell (Treg) imbalance is associated with COPD	2022
34 (38)	eosinophile granulocyte	Tissue-infiltrating eosinophils, basophils, and eosinophils in the lungs of COPD patients promote immune mechanisms	2020
35 (39)	Lung microbiota	Association between Pseudomonas abundance and chronic respiratory diseases	2024
36 (40)	Tuberculosis bacilli	Mechanisms of pulmonary immunity induced by M. tuberculosis	2023
37 (41)	Airway microbiota	Immunophenotypic airway microbiota can advance COPD, with immunotherapy	2022
38 (42)	Adaptive immune cells	Adaptive immune cells may play a key role in the COPD pathophysiology.	2023
39 (43)	Streptococcus pneumoniae	Innate immune stimulation mediated by TLR-5 may ameliorate the development of bacteria-induced exacerbations in the COPD setting	2020
40 (44)	The innate immune system	Bronchial epithelial cells, neutrophils, macrophages, and other cells exhibit multiple impaired immune mechanisms	2022
41 (45)	Neutrophil elastase	The NE is involved in lung destruction	2020
42 (46)	Human neutrophil elastase (HNE)	Ability to release a wave of proteolytic activity by inhibitors that disrupt other proteases	2021
43 (47)	Macrophage	Macrophages secrete inflammatory cytokines/chemokines and activate transcription factors in the pathogenesis of inflammatory lung disease	2021
44 (48)	Immune-inflammation	Monocytes, mast cells, and alveolar type 2 cells (AT2 cells) are implicated in the etiology of COPD	2023
45 (49)	Polysaccharides and the immune response	The effect of BSP on COPD is related to the abundance of intestinal microbiota and explored the underlying mechanisms	2024
46 (50)	Respiratory Epithelial Alert protein (IL-33)	IL-33 causes lesions by inducing autoantibodies against lung tissue, especially alveolar type II epithelial cells	2019
47 (51)	Upper respiratory tract (URT) microbiome	Interaction of the lung microbiome with the oropharyngeal and gut microbiome, emphasizing its role in both innate and adaptive immune responses	2024
48 (52)	COPD and Lung Squamous Cell Carcinoma (LUSC)	HTR 2 B, DPYS, FRY, and CD19 are the key genes for COPD	2024
49 (53)	Immunological cell	Innate lymphocytes (ILC 3), and dendritic cells (DC) are involved in chronic obstructive pulmonary disease (COPD)	2024
50 (54)	Self-immunprocess	Elucidate the relationship with autoimmunity in patients with stable COPD	2020
51 (55)	Immunosignaling pathway	The cyclic GMP-AMP synthase stimulator (cGAS-STING) signaling pathway of the interferon gene plays an important new role in the immune system	2024
52 (56)	CircRNA	Increasing proportion of M2 macrophages in lung tissue in humans and mice with smoking-associated emphysema	2024
53 (57)	Characteristic genes and immune cell infiltration	Characteristic genes GPC 4 and RS1 are regulated by miR-374a-3p	2022
54 (58)	Neutrophile granulocyte	Novel role of neutrophils in lung biology, the molecular mechanisms controlling neutrophil transport to the lungs	2021
55 (59)	The macrophages of the innate immune system	The importance of macrophages in orchestrating immune responses, interacting with other cells, and the ability to exhibit dysregulation	2023
56 (60)	Cell senescence	EP300, MTOR, NFE2L1, TXN key genes in cellular senescence and COPD progression relationship	2024
57 (61)	Cell factor	The interleukin-1 (IL-1) family of cytokines and receptors is associated with the function of innate and adaptive immunity and the development of inflammation.	2022
58 (62)	Alveolar macrophage	The role of AM in the protective effect of angiotensin II type 2 receptor (AT2R) activation on cigarette smoke (CS) -induced COPD	2022
59 (63)	Immune-related genes	The number of resting NK cells, activated DCs, and neutrophils in the lung tissue of COPD patients was higher than normal	2022
60 (64)	Exogenous signaling pathways	Hyperactivated PI3K signaling plays a crucial role in COPD pathogenesis	2021
61 (65)	Neutrophil extracellular traps (NETs)	Probably reprogram metabolism to regulate immune function, the research of immune metabolism has surged	2022
62 (66)	Immune cells in the tertiary lymphoid organ (TLO)	The role of lung dendritic cells (DC) in the induction of follicular helper T cell cells (Tfh) cells in COPD	2020
63 (67)	The innate immune response	Of TSLP, IL-33, IL-25, and HMGB1 in the pathogenesis of asthma, COPD, idiopathic pulmonary fibrosis, and cystic fibrosis	2023
64 (68)	Immune network and factors	AE-COPD is driven by the transport of immune cells to the lungs, along with the upregulation of the expression of specific chemokines in the lung tissue	2019
65 (69)	Peripheral nervous system	Existing data on the association between COPD and polyneuropathy, including possible pathophysiological mechanisms	2022
66 (70)	Ascariasis	Larvae result in prolonged and sustained lung tissue damage	2024
67 (71)	Innate immune cells	Possible mechanism linking COPD and both periodontitis diseases in terms of innate immune cells	2022
68 (72)	HIV circadian dysregulation mechanisms	Circadian genes play a crucial role in lung pathology, especially in PLWH.	2023
69 (73)	Immune parameter levels	Levels of certain inflammatory and immune parameters, including C-reactive protein (CRP) and circulating immune complex (CIC) and the CRP/CIC index, were assessed in COPD patients	2019
70 (74)	Cadmium (Cd)	Contact to cadmium is associated with the development and deterioration of chronic lung diseases such as pulmonary fibrosis and COPD	2024
71 (75)	Macrophage	The functional importance of macrophages in lung tissue homeostasis and inflammation, and highlights how environmental factors alter macrophage plasticity and function in the lung airways	2019
72 (76)	Immunologic function	The critical role of the immune system in COPD and its therapeutic potential	2024
73 (77)	Airway epithelial structure and function	How the abnormal epithelial structure and function caused by chronic injury and abnormal repair can contribute to airway disease	2023
74 (78)	Microbial dysbiosis crosstalk	Interaction or crosstalk between the lung and the gut, and how such interactions or crosstalk are affected or influenced by the microbiota	2020
75 (79)	Immunological cell	This study found a potential causal relationship between immune cells in COPD	2024
76 (80)	Natural killer (NK) cells	In subsets in peripheral blood and lung, highlighting the important role that NK cells play in COPD and its deterioration	2021
77 (81)	Adaptive immunity	Local disruption of the mucosal immune barrier of secretory immunoglobulin A (SIgA) is associated with the accumulation of lymphocytes in the airway wall and the development of tertiary lymphoid structures (TLS) around the small airways	2021
78 (82)	The alveolar tissue was lost	IL-33 and its receptor ST2 mediate immune responses and alveolar injury, and their expression is upregulated in COPD patients, which is associated with disease progression	2023
79 (83)	Immunogene	On how innate immune signaling from gene expression to splicing is altered upon *in vivo* exposure to CS, and highlights an important novel role for lincRNA-Cox 2 in the regulation of immune genes following smoke exposure	2023
80 (84)	Airway mucus	Chronic mucus overload (CMH) is a major factor driving the increased risk of morbidity and mortality in specific subsets of COPD patients	2023
81 (85)	Caducity	Summarizes findings on how aging changes immunity to respiratory diseases to identify age-affected pathways and mechanisms leading to the development of lung disease	2023
82 (86)	Histone deacetylase (HDAC)	Inhibition of HDACs is a prospective therapeutic approach that reverses epigenetic alterations in several diseases	2022
83 (87)	Th 17	The influence mechanism of TLR 2 and TLR 4 expression, to investigate the contribution of TLR to the development of the helper T cell (Th) immune response in COPD	2020
84 (88)	T helper cells (Th)	Enhanced TLR 2 expression on CD4 cells shifts the cytokine profile to a Th 17 phenotype, which plays a crucial role in COPD progression	2021
85 (89)	Airway mucin	Airway mucin 5AC (MUC5AC) and MUC5B concentrations are increased during exacerbations of spontaneous and experimental-induced chronic obstructive pulmonary disease	2022
86 (90)	Eosinophile granulocyte	COPD patients with a higher eosinophil count were associated with an increased clinical response to a phosphodiesterase-4 inhibitor (PDE 4 i)	2021
87 (91)	Intestinal microecology	How the gut-lung axis affects COPD, including changes in the intestinal microecology, pathological mechanisms, and the immune responses involved	2024
88 (92)	Common pollutants	COPD stage-specific genetic alterations in basal cells that affect bronchial ascending branch cell composition and may control disease-specific epithelial elastic mechanisms targeting environmental nanoparticles	2024
89 (93)	Alveolar macrophage	Less expression of Siglec-1 in alveolar macrophages of COPD patients, and Siglec-1 participates in the phagocytosis of NTHi	2020
90 (94)	Neutrophil extracellular trap (NET)	Interplay of NET in COPD development and pathophysiology	2021
91 (95)	Immunoglobulin	Immunoglobulin therapy may be effective in preventing recurrent AECOPD	2021
92 (96)	Immunophenotyping of lung myeloid cells	In COPD patients, there were marked differences in the abundance of PDL 1 (high) and PDL 2 (high) clusters, and in PDL 1 and PDL 2 expression in several macrophage subtypes	2020
93 (97)	Cell senescence	Aging of macrophages (especially alveolar macrophages) plays a crucial role in the pathophysiology of COPD	2024
94 (98)	Neutrophil extracellular traps (NETs)	The NADPH oxidase, ERK 1/2, and p38 MAPK signaling pathways are implicated in the formation of Cd-induced NETs	2019
95 (99)	Of ceRNA, and the coexpression network	Different proportions of immune cells affect the changes in the size of lung function parameters	2023
96 (100)	exosome	Exosomes accelerate the progression of chronic obstructive pulmonary disease (COPD) by upregulating the trigger receptor (TREM-1) expressed on myeloid cells 1	2024
97 (101)	immunocyte	Macrophages, alveolar macrophages, and T cells are involved through immunity and inflammation	2022
98 (102)	immunocyte	A variety of immune cells, intervene in inflammation and immune related pathways	2021
99 (103)	alveolar macrophage	The differentially expressed genes in the AMs of COPD patients are related to the molecular mechanisms of immunity and inflammation	2020
100 (104)	Respiratory tract mucosal immunity	Clarify the mechanism of action from the perspective of respiratory mucosal immunity	2020
101 (105)	macrophage	Coculture of the human macrophage cell line THP-1 inhibited ciliary protein (β -tubulin-IV) levels in BEAS-2B cells	2021
102 (106)	CCAAT Enhancer-binding protein β (C/EBP β)	Regulation of C/EBP β and related signaling pathways in chronic obstructive pulmonary disease	2022
103 (107)	Alterations in alveolar structure	The acidic and cysteine-rich (SPARC) -containing secreted proteins that bind collagen are involved in COPD	2022
104 (108)	Pyroptosis and immune imbalance	Effects of pyroptosis and immune imbalance mediated by high mobility group protein B1 (HMGB 1) in the pulmonary arterial hypertension (COPD-PH) associated with chronic obstructive pulmonary disease	2023
105 (109)	Immune cell infiltration, and metabolism	Suggesting that reactive oxygen species (ROS) play an important role in COPD with MetS	2024
106 (110)	immunocyte	Interaction between the immune system and the lungs	2024
107 (111)	Modification of m6A	Role and related mechanisms of the m6A methyltransferase ZC3H13 in COPD.	2024
108 (112)	T cellular level	Effect of altered T cell levels on immune function	2019
109 (113)	Congenital lymphocytes (ILC)	How ILC 3 acts on pathogen challenge and lung inflammation, and the underlying mechanisms	2021
110 (114)	Immune properties and transcriptional regulation mechanisms	Nine miRNA and three TFs target MTHFD2, GFPT2, PHLDA1 and FGG to be associated with COPD	2021
111 (115)	Human lung tissue-resident memory T cells (T (RM))	Recent developments in the creation and maintenance of T (RM) cells in the lung, describing their role in COPD	2021
112 (116)	Epigenetic modification	Epigenetic modifications activate macrophages to participate in COPD	2024
113 (117)	hydrone	The miR-194-3p, miR-502-5p, and miR-23a-5p may be involved in the pathogenic mechanism of COPD	2021
114 (118)	T cell	CD4 T-cell viability, T-helper T-cell 17 (Th 17)/regulatory T (Treg) markers, and CXCR4 expression in T cells or spleen/lung tissue	2023
115 (119)	macrophage	Soluble extracts can trigger the death of respiratory macrophages, impairing their phagocytic capacity	2022
116 (120)	LncRNA	LncRNA LUCAT1 May be an important indicator to identify COPD	2021
117 (121)	Macrophage polarization	Macrophage polarization may be useful in the COPD diagnosis	2021
118 (122)	Lung microbiome	Microbiome composition, microbial metabolites, and interactions between the lung microbiome and host immunity have profound effects on COPD	2023
119 (123)	The immune system cells	DPP-4 is expressed in the cells of the immune system; it has a wide range of biological functions in immune regulation, cancer biology, and glucose metabolism	2020

**Table 2:** Research articles related to immunization and COPD.

Number	Machine	Model	Norm	Result
1	Innate lymphocytes (ILC)	COPD rat	Poor spirometry, sputum inflammation, and GATA3,ILC (ILC 2)	The presence of sputum ILC 2 is predictive of COPD severity and reveals a new pathway of IL-17A plasticity in lung ILC 2 that is prevented by the immunomodulatory protein SP-D
2	Deiodinase type 2 (DIO 2), chemokines	COPD patient	Serum indicators	DIO 2 levels were higher in COPD patients than in controls
3	Exposure to air pollution of PM is associated with COPD incidence	COPD rat	MHC I, antigen presentation, and the adaptive immune response	GATA 3/lncRNA MHC-R regulated immune activity, involved In air pollution PM induces COPD pathogenic mechanisms
4	Macrophage number and phagocytosis	COPD patient	Effects of lung macrophage phenotype and function	Corticosteroids play therapeutic roles in COPD
5	Iron-loaded macrophages	COPD patient	The sputum contained an index hemosiderin index	IL-6-dependent iron sequestration by sputum macrophages may lead to immune cell dysfunction
6	Actin-associated protein (Arp) 2/3 complex	COPD rat	AM phagocytosis function	PM2.5 exacerbates AM phagocytosis in COPD mice by inhibiting the Arp 2/3 complex and F-actin
7	Inductive bronchial-associated lymphoid tissue (iBALT)	COPD rat	Lung pathology	The formation of iBALT and sequestration of effector T cells may be the immune system reducing lung inflammation
8	Unbalance between pro-inflammatory and anti-inflammatory immune responses	COPD rat	The expression of FOXP3 and IL-10 et al	Microenvironmental stimuli produced by cytokine release during COPD progression cause a Th 17/Treg imbalance
9	Dysbiosis of the gut and lung microbiota and COPD	COPD rat	Th 17 cells, Treg cells, and their associated cytokines, transcription factors, as well as the gut and lung microbiota	Clearing the lung and replenishing the blood may play a therapeutic role by maintaining the balance of immune cells and regulating the gut and lung axial flora
10	Eosinophile granulocyte	COPD patient	Anatomical distribution pattern of infiltrating eosinophils	The identification of a spatially-limited eosinophil-rich type 2 microenvironment represents a novel heterogeneity in COPD immunopathology
11	Lung microbiota	COPD patient	Effect of mononas on epigenetic modifications and their relationship to lung disease	Pseudomonas may influence cellular mechanisms through epigenetics to facilitate the development and progression of chronic respiratory diseases.
12	Monoplast RNA	COPD patient	Three single-cell RNA sequencing datasets for COPD	Single-cell-level mechanisms of COPD pathogenesis
13	Bletilla striata polysaccharide	COPD rat	*in vitro* study	BSP, attenuated the inflammatory response and inflammatory cell infiltration in the lung tissues of COPD mice
14	Airway inflammation, remodeling, and alveolar structural damage	COPD patient COPD rat	Immunstudies with IgG autoantibodies	Serum autoantibodies produced by mice bind not only to mice but also to human alveolar type II epithelial cells
15	Innate lymphocytes (ILC 3), and dendritic cells (DC)	COPD rat	*In vivo* and *in vitro* experiments	DC activation induced by CS enhanced the differentiation of ILC to NCR (-) ILC 3
16	Airway and systemic autoantibody responses	COPD patient	Sputum supernatant and serum	There was a separation between sputum autoantibodies and serum autoantibodies in patients with stable COPD
17	AM	COPD rat	AM polarization, phagocytosis, and metabolism	Alveolar macrophages (AM) are the first immune defense line of the respiratory system and play a key role in lung homeostasis
18	Larval antigen and infection	COPD rat	The immunohistochemical method	Ascaris antigen induces a chronic immune response in the host lungs after larval migration
19	Inflammation and immunity	COPD patient	Blood serum	The action of non-specific inflammatory mechanisms may be enhanced in the more advanced COPD stage
20	The T lymphocyte response	COPD patient And the COPD + SIgA-deficient mice	Small airway	Endogenous bacteria coordinate persistent pathological T gonorrhea through monocyte recruitment and differentiation
21	T helper cells (Th), immunoreactive	COPD patient	Blood serum	Endogenous bacteria coordinate persistent pathological T gonorrhea through monocyte recruitment and differentiation
22	Alveolar macrophage	COPD patient	Cell suspensions were collected in the lung tissue	The pression of Siglec-1 is reduced in alveolar macrophages of COPD patients
23	Exosome	COPD mouse	*In vitro* and *in vivo* experiments	CSE, treated MAECs-derived exosomes for M1 macrophage polarization and cell pyroptosis in COPD
24	Immune cell subsets in the lung tissue	COPD patient COPD rat	Lung function, lung pathology, and blood serum	Liuwei Buqi capsule reduces lung function, lung damage and other tissue pathology
25	Respiratory tract mucosal immunity	COPD rat	Immunohistochemical staining and flow cytometry	It can enhance the immune function of the respiratory tract
26	Airway remodeling and macrophages	COPD mouse	*In vitro* experiments and *in vivo* experiments	High expression of BMP-2 aggravates the development of COPD
27	The m6A-methylated transferase ZC3H13	COPD mouse	Serum, lung tissue, etc	Targeting ZC3H13/ITGA6 may be a potential therapeutic approach for treating COPD
28	T cellular immune function	COPD patient	Mortality, blood gas analysis, acute physiology and chronic health assessment	Altered effects on immune function based on T cell levels
29	T lymphocytes (ILC)	COPD patient	Cell proliferation and apoptosis assays	LncRNA LUCAT1 May be an important indicator to identify COPD

## 2 Innate immunity and COPD: cells and related mechanisms

Innate immunity refers to the defense mechanisms that the body is born with, including the body’s physical barriers, nonspecific immune cells and serum proteins, and other nonspecific defense factors, which in the progression of COPD include cellular pattern recognition, surface barriers, cellular components, inflammation, and the complement system. The innate immune system employs specialized germline-encoded detectors called pattern recognition receptors (PRRs) to identify pathogenic signatures. These transmembrane or cytoplasmic proteins function as molecular surveillance systems, primarily expressed on sentinel immune cells including dendritic cells, macrophages, monocytes, neutrophils, and epithelial barriers (17). PRRs demonstrate dual specificity by recognizing: 1) Pathogen-associated molecular patterns (PAMPs) - conserved microbial components essential for pathogen survival, and 2) Damage-associated molecular patterns (DAMPs) - endogenous molecules released during tissue injury or necrotic cell death (124).

**Figure 2:**
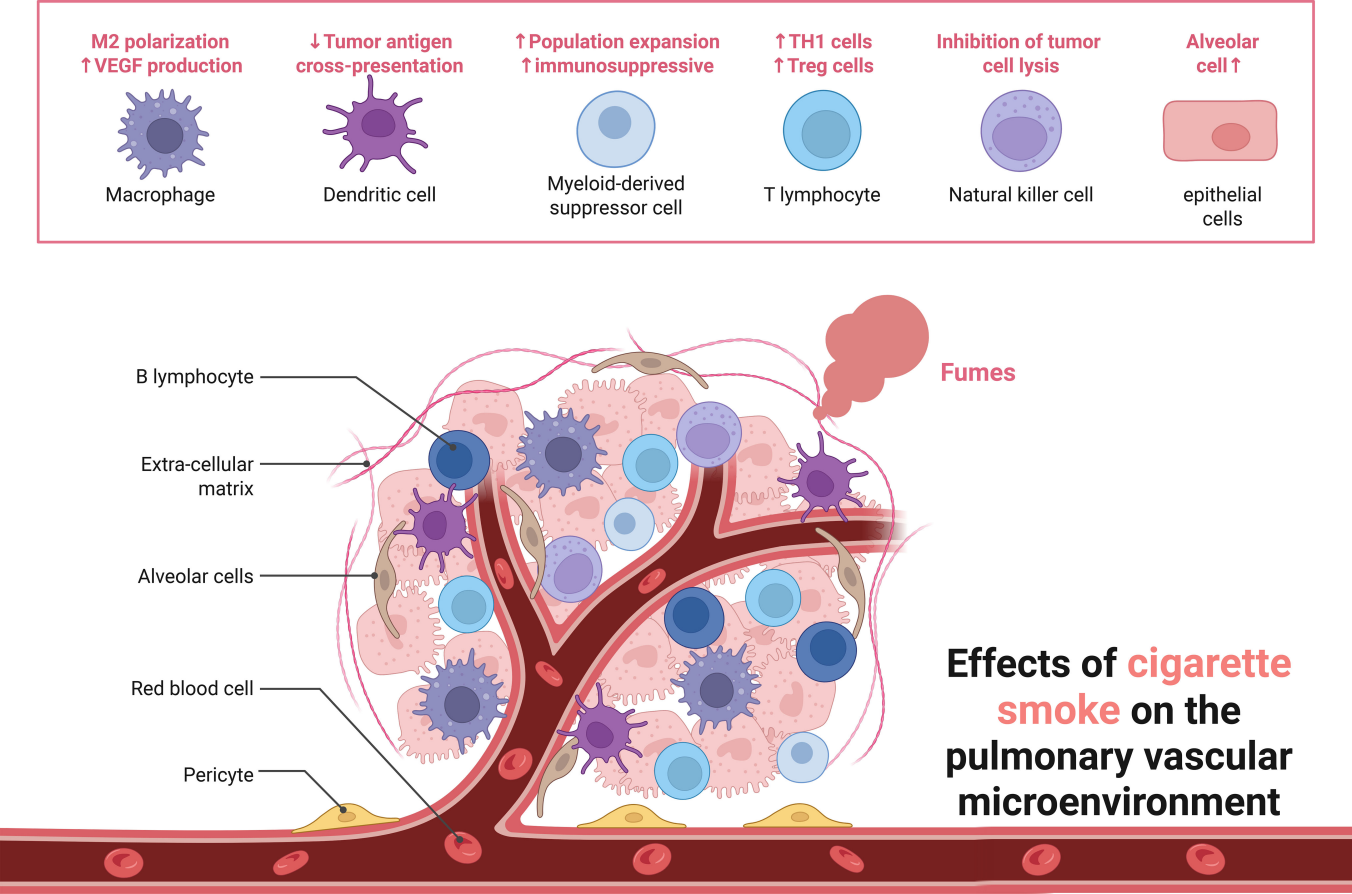
Effects of cigarette smoke on the pulmonary vascular micro environment.

Among the cellular pattern recognition covered, surface barrier studies focus on COPD with gut flora, lung-gut axis, and cellular components including macrophages, neutrophils, dendritic cells and innate lymphocytes. Macrophages are one of the major sources of cytokines and inflammatory mediators in the airways, and in addition to phagocytosis of particles, bacteria, and apoptotic cells, the production and secretion of inflammatory mediators promotes the accumulation of other inflammatory cells, such as neutrophils. Neutrophils migrate from the pulmonary capillaries to the airways and kill pathogenic microorganisms (fungi, bacteria, viruses, etc.) by means of reactive oxygen species, antimicrobial proteins, and degradative enzymes (e.g., elastase). Overproduction, release, and inadequate neutralization of these potentially tissue-damaging molecules have been shown to contribute to tissue destruction in COPD. In addition, these infiltrating inflammatory immune cells can activate adaptive immune responses by modulating antigen presentation by antigen-presenting cells in lung tissue, including dendritic cells and macrophages (**Figure 2**).

### 2.1 Macrophage

AMs play a central role in processing and clearing lung particles in COPD patients. Increased recruitment of monocytes in response to monocyte-selective CCL2 and CXC chemokine ligand 1 (CXCL1) further leads to increased levels of macrophages in sputum and bronchoalveolar lavage fluid (BALF) of COPD patients. And sustained exposure to cigarette smoke or biomass significantly depleted intracellular antioxidants, such as glutathione, leading to excessive oxidative stress, which inhibited bacterial phagocytosis and cytophagocytosis in AMs. As a result, AMs in COPD produce more pro-inflammatory factors, leading to tissue damage, as well as defective phagocytosis of AMs, which together influence the progression of COPD (125). Macrophages are highly plastic and can differentiate into distinct phenotypes based on microenvironmental signals, primarily including pro-inflammatory M1 and anti-inflammatory M2 phenotypes. In COPD, macrophage plasticity is regulated by multiple factors, such as transcription factors, signaling pathways, and epigenetic modifications. Studies demonstrate that the polarization state of alveolar macrophages directly influences both the inflammatory response and tissue remodeling in COPD (126). Additionally, macrophages contribute to COPD-associated airway inflammation and fibrosis through regulation of inflammatory mediator release and extracellular matrix metabolism (127).

Macrophage plasticity and polarization status constitute pivotal determinants of COPD pathogenesis. Classically activated M1 macrophages, typically induced by pro-inflammatory stimuli (e.g., LPS and IFN-γ), perpetuate airway inflammation through robust secretion of inflammatory mediators including TNF-α, IL-1β, and IL-6. Conversely, alternatively activated M2 macrophages, polarized by anti-inflammatory cytokines (IL-4/IL-13), actively contribute to tissue remodeling processes by promoting fibrosis and repair mechanisms. Critically, the imbalance between pro-inflammatory M1 and pro-fibrotic M2 phenotypes drives progressive pulmonary destruction in COPD. Polarized M1 and M2 macrophages are also involved in the pathogenesis of COPD: the phagocytic capacity of M1 macrophages and the antigen-presenting properties of Th1 produce a variety of Th1 cytokines (128), e.g.IL-1β, IL-6, IL-12, and TNF-α. It has been found that the activation of the Notch signaling pathway in the cigarette-induced model of COPD and the further polarization of macrophages shifted toward the M1 phenotype with increased levels of IL-6, TNF-α and reactive oxygen species. In contrast, in the face of foreign particulate matter and microbial exposure, M2 macrophages induced by Th2 cytokines overexpressed arginase-1 (Arg-1), IRF4, and mannose receptor CD206 and released TGF-β, leading to myofibroblast activation, smooth muscle proliferation, and aberrant tissue repair. Another study found that e-cigarette exposure enhanced airway dilation, mucus secretion and fibrogenesis in COPD mice, which was associated with an increased M2 macrophage phenotype (79).

### 2.2 Congenital intrinsic lymphoid cells

Innate lymphoid cells (ILC) are a group of innate immune cells that are derived from common lymphoid progenitor cells (CLP) and belong to the lymphoid lineage. There are no antigen-specific B or T cell receptors to define these cells due to the lack of recombination activation genes (RAG). ILCs do not express myeloid or dendritic cell markers (129).

Natural killer cells, one of the member ILCs (130), are lymphocytes that are part of the innate immune system and do not directly attack invading microorganisms. Instead, NK cells destroy damaged host cells, such as tumor cells or virus-infected cells, and recognize such cells through a condition known as the “missing self”. The term describes low levels of the cell surface marker MHC I (major histocompatibility complex), which can occur in viral infections of host cells. They were called “natural killers” because the original concept was that they did not need to be activated to kill cells that “lacked self”. For many years, it was not clear how NK cells recognized tumor cells and infected cells. It is now known that the MHC composition on the surface of those cells is altered and NK cells are activated by recognizing the “missing self”. Normal human cells are not recognized and attacked by NK cells because they express their own MHC antigens intact. Those MHC antigens are recognized by the killer cell immunoglobulin receptor (KIR), which actually puts the brakes on NK cells (13, 131).

In a study on the phenotyping and quantification of human lung ILC subpopulations (132), the investigators set up control and COPD groups with the presence of COPD as a variable, and identified ILC subpopulations in digested human lungs by flow cytometry, further defining subsets based on specific surface markers, and found that in the control group, both ILC2 and NCR-ILC3 were the most abundant ILC subpopulations; whereas in the COPD group, the lung ILC subpopulation was more heavily weighted toward NCR- ILC3.Although all 3 ILC subpopulations have been identified in the airways, most studies to date have focused on ILC2, emphasizing their importance in tissue repair, protection against helminthic infections, and involvement in a wide range of allergic diseases. However, ILC3 is actually the most common ILC subpopulation in human lung tissue.ILC3 and LTi cells rapidly secrete IL-17 and IL-22, driving the development of COPD (80).

In mice, cigarette smoke exposure leads to an increase in all ILC subpopulations in bronchoalveolar lavage fluid, particularly IFN γ+ and IL-17+ ILC.IL-17 is considered a key driver of neutrophil inflammation in COPD (133), inducing neutrophil maturation and recruitment, and through the release of its proteolytic enzymes (elastase, histone G, and protease-3), facilitating Sputum IL-17 is higher in COPD patients than in non-smoking controls, and bronchial biopsies from COPD patients also show increased expression of IL-17, IL-22 and IL-23. ILC3-secreted GM-CSF is critical in allergic airway disease, human lung anti-microbial defense, and homeostasis of alveolar surfactant. ILC1, Th1 cells and CD8+ cells are essential in the development of allergic airway disease. ILC1, Th1 cells and CD8+ T cells can produce IFN γ, which is involved in the pathogenesis of COPD by inducing the production of elastase and nitric oxide by alveolar macrophages, leading to emphysema (134).

### 2.3 Dendritic cells

Dendritic cells (DCs) are phagocytes in tissues that come into contact with the external environment. In the lungs, dendritic cells exist in multiple subpopulations, including myeloid dendritic cells (mDCs) and plasmacytoid dendritic cells (pDCs). mDCs primarily mediate T cell-mediated immunity and antibody production, while pDCs play a crucial role in antiviral immunity and immune tolerance (114).In COPD patients, the numbers and functions of these subpopulations undergo significant changes. Studies have shown that in the small airways and alveoli of COPD patients, the number of immature dendritic cells significantly increases, while the number of mature dendritic cells decreases, particularly in advanced COPD patients, where alveolar CD1a+langerin-, BDCA-2+CD11c+dendritic cell subpopulations significantly increase (135).These changes are closely associated with the immunopathology of COPD. Plasmacytoid dendritic cells (pDCs) accumulate significantly in pulmonary lymphoid follicles of COPD patients, especially in those with mild to moderate COPD (136). This accumulation may be associated with viral infections and autoimmune responses in COPD. Additionally, pDCs exhibit enhanced secretion of TNF-α and IL-8 in COPD, further exacerbating inflammatory responses (137).

**Figure 3:**
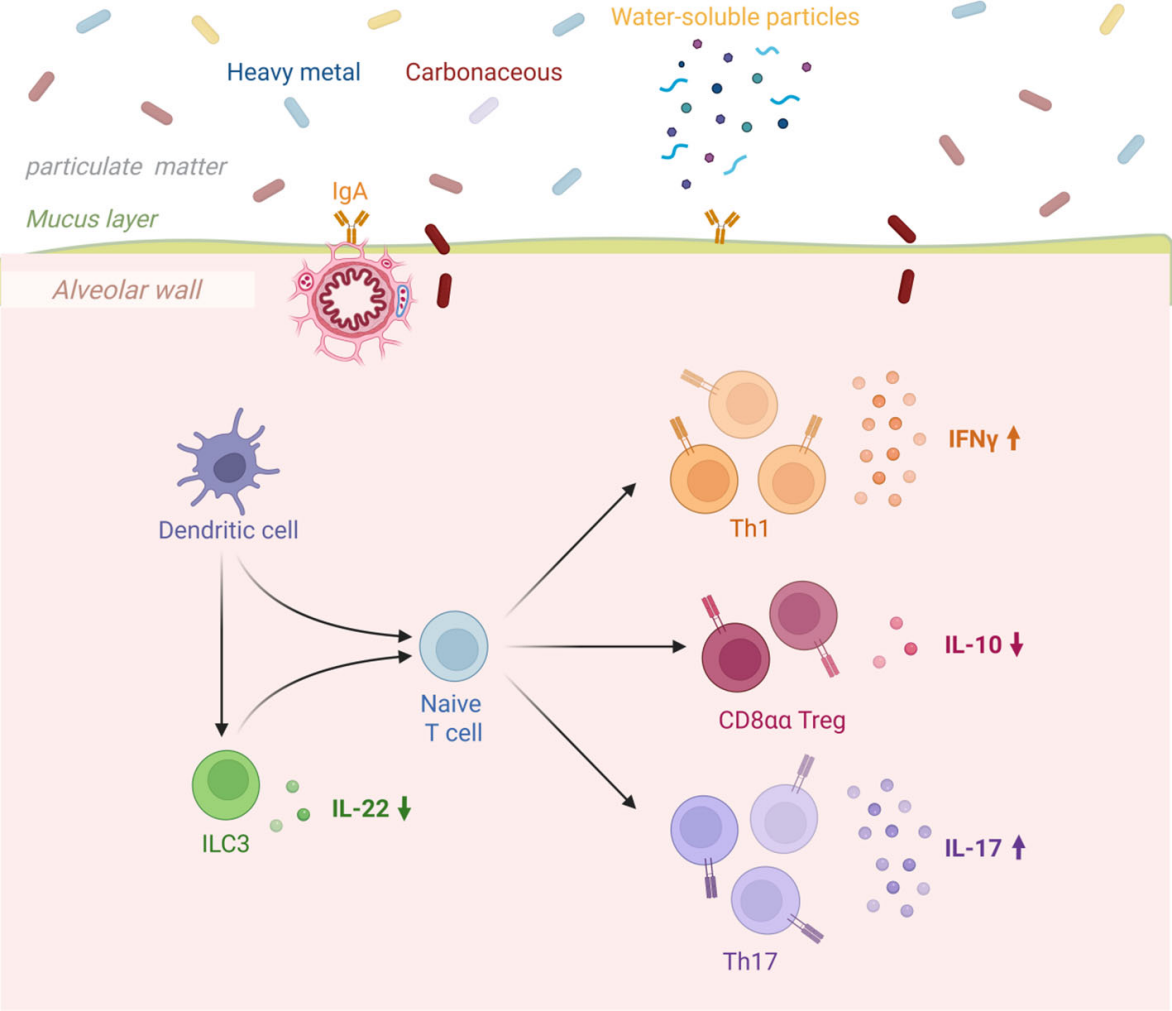
Congenital intrinsic lymphocytes in relation to COPD.

Dendritic cells serve as a critical bridge between tissues and the immune system, coordinating immune responses by presenting antigens to T cells. Studies have shown that cigarette smoke-induced dendritic cell activation in chronic obstructive pulmonary disease (COPD) promotes the differentiation of ILCs into NCR-ILC3, thereby influencing disease severity (26). These cells initiate immune responses by recognizing pathogen-associated molecular patterns (PAMPs) and damage-associated molecular patterns (DAMPs) through Toll-like receptors (TLRs) (2).In the progression of COPD, dendritic cells exhibit four major functional dysregulations: First, their antigen-presenting function is inhibited, and exposure to cigarette smoke impairs cell maturation and cytokine secretion, weakening immune defense against bacteria such as Streptococcus pneumoniae (13); second, TLR2-activated dendritic cells drive Th17 cell differentiation, promoting emphysema formation and chronic inflammation; third, the immune tolerance mechanism mediated by dendritic cells is impaired, leading to reduced regulatory T cell (Treg) responses and triggering excessive inflammation and autoimmune reactions; additionally, oxidative stress further disrupts dendritic cell function, accelerating the pathological process (11).These functional dysregulations collectively cause T cell response imbalance, closely associated with airway remodeling and persistent inflammation in COPD (**Figure 3**).

Dendritic cells recognize pathogen-associated molecular patterns (PAMPs) and damage-associated molecular patterns (DAMPs) through Toll-like receptors (TLRs), thereby initiating an immune response (17).In COPD, the antigen-presenting function of dendritic cells is impaired, leading to an imbalance in T cell responses (138), in COPD, dendritic cells are activated via TLR2, promoting the differentiation of Th17 cells and contributing to the development of emphysema. This Th17 response is closely associated with the chronic inflammation and airway remodeling characteristic of COPD. Additionally, oxidative stress further exacerbates the pathological process of COPD by impairing dendritic cell function. Impaired tolerance function of dendritic cells leads to excessive inflammatory and autoimmune responses, characterized by reduced regulatory T cell (Treg) responses, further exacerbating immune imbalance (139).

### 2.4 Granulocyte

Granulocytes are white blood cells that have granules in their cytoplasm. In this category are neutrophils, mast cells, basophils and eosinophils. Mast cells reside in connective tissue and mucous membranes and regulate inflammatory responses. They are most often associated with allergies and allergic reactions. Basophils and eosinophils are related to neutrophils. They secrete chemical mediators that defend against parasites and play a role in allergic reactions such as asthma (140). Eosinophils and basophils have been studied more in COPD related studies (38), one study on the anatomical distribution pattern of infiltrating eosinophils in COPD patients found a novel heterogeneity in the immunopathology of COPD patients, which manifested itself in the identification of a spatially restricted eosinophil-rich type 2 microenvironment, and another study focusing on eosinophils demonstrated that patients with COPD Tissue-infiltrating eosinophils, basophils, and eosinophil-promoting immune mechanisms in the lungs (141).

**Figure 4:**
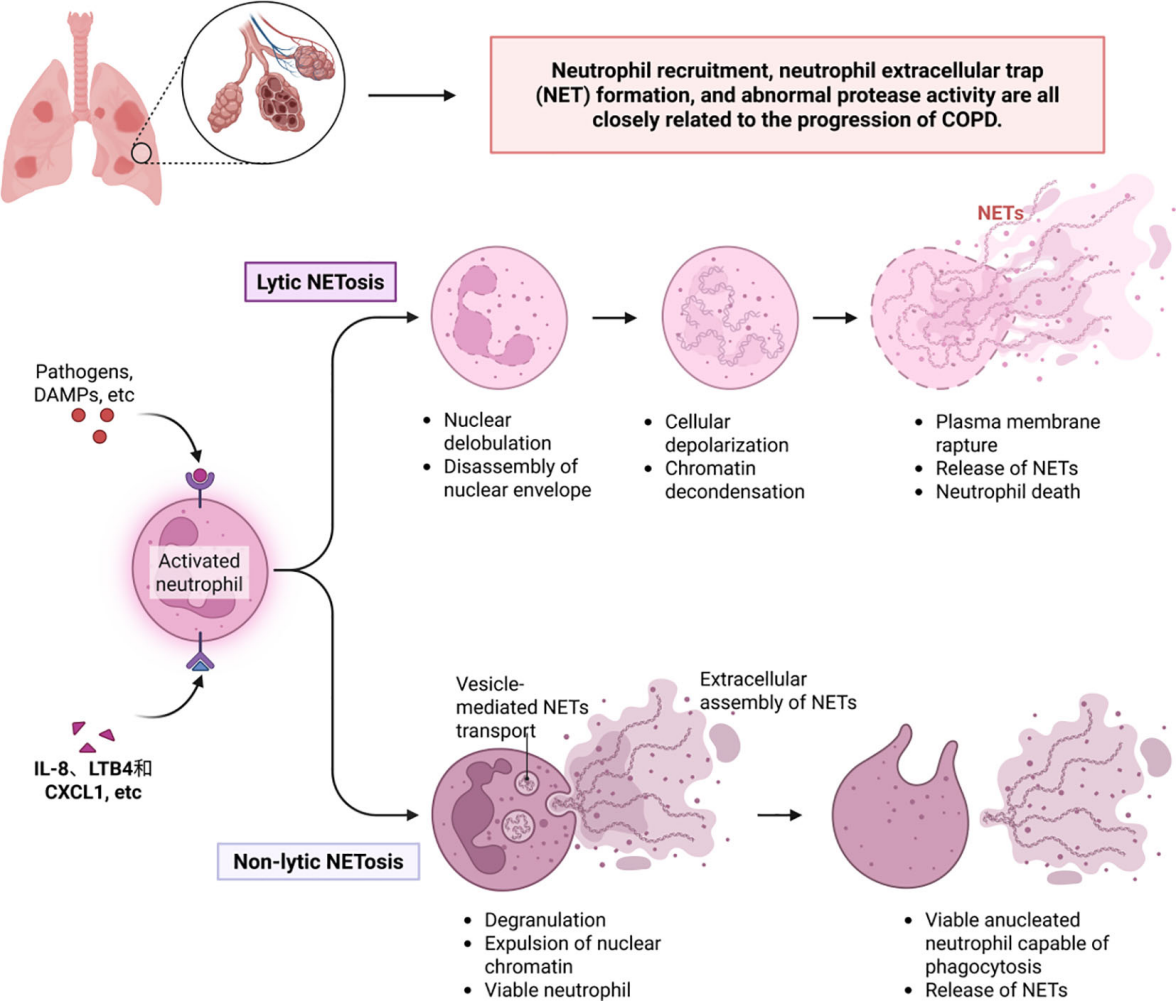
Neutrophil extracellular traps and COPD.

It is worth noting that under the regulation of multiple inflammatory factors, oxidative stress, and hypoxic environments, neutrophils play a key role in the pathogenesis of COPD. Abnormalities in neutrophil recruitment, neutrophil extracellular trap (NET) formation, and protease activity are closely associated with disease progression (142) (**Figure 4**). First, inflammatory mediators such as IL-8, LTB4, and CXCL1 activate endothelial cells, mediating neutrophil migration to the airways. This process involves the release of chemokines by lung epithelial cells and macrophages, prompting neutrophils to interact with endothelial cells via adhesion molecules such as CD11b/CD18, ultimately crossing the vascular wall to infiltrate lung tissue (143).Infiltrating neutrophils release reactive oxygen species (ROS) and proteases, directly damaging lung structures and exacerbating inflammatory responses. Neutrophil extracellular traps (NETs) exhibit dual effects in COPD: on one hand, their DNA-histone-antimicrobial protein complexes can capture pathogens, providing protective effects against infections during acute exacerbations; on the other hand, excessive NET formation releases histones and proteases that damage alveolar epithelial and endothelial cells and exacerbate airway inflammation and fibrosis by activating inflammasomes and promoting cytokine release, ultimately leading to lung tissue damage (144). Proteases released by neutrophils (including elastase, matrix metalloproteinases, and cathepsins) are core pathological mediators, elastase directly degrades elastic and collagen fibers in alveolar walls, promoting the formation of pulmonary emphysema; these proteases amplify the inflammatory cascade by activating inflammatory mediators such as IL-8 and TNF-αand disrupting theα1-antitrypsin barrier; simultaneously, they induce airway smooth muscle proliferation and fibrosis, leading to airway remodeling and progressive airflow limitation (145).

Neutrophilic COPD is the primary phenotype of COPD and differs fundamentally from eosinophilic COPD (accounting for 20–40%) (146). The eosinophilic subtype is characterized by a Th2 immune response, releasing IL-5/IL-13 and eosinophil cationic protein. Clinically, it presents with mild airflow limitation but responds well to glucocorticoid therapy. Elevated eosinophil counts in blood or sputum indicate a risk of acute exacerbation and a favorable prognosis; the neutrophilic type mediates tissue destruction through elastase, MMP-9, and IL-8/IL-17, presenting with severe airflow obstruction, high sputum volume, and hypoxemia. Patients are resistant to corticosteroids and require bronchodilators/antibiotics. Elevated neutrophil/lymphocyte ratios and C-reactive protein (CRP) levels predict high hospitalization rates and mortality (141, 147). Microbiome analysis shows that the eosinophil-dominant phenotype is associated with an enrichment of the genus Campylobacter, whereas the neutrophil-dominant phenotype is dominated by the genus Haemophilus and accompanied by elevated IL-1β/TNF-α levels (148).

### 2.5 Epithelial cells

**Figure 5:**
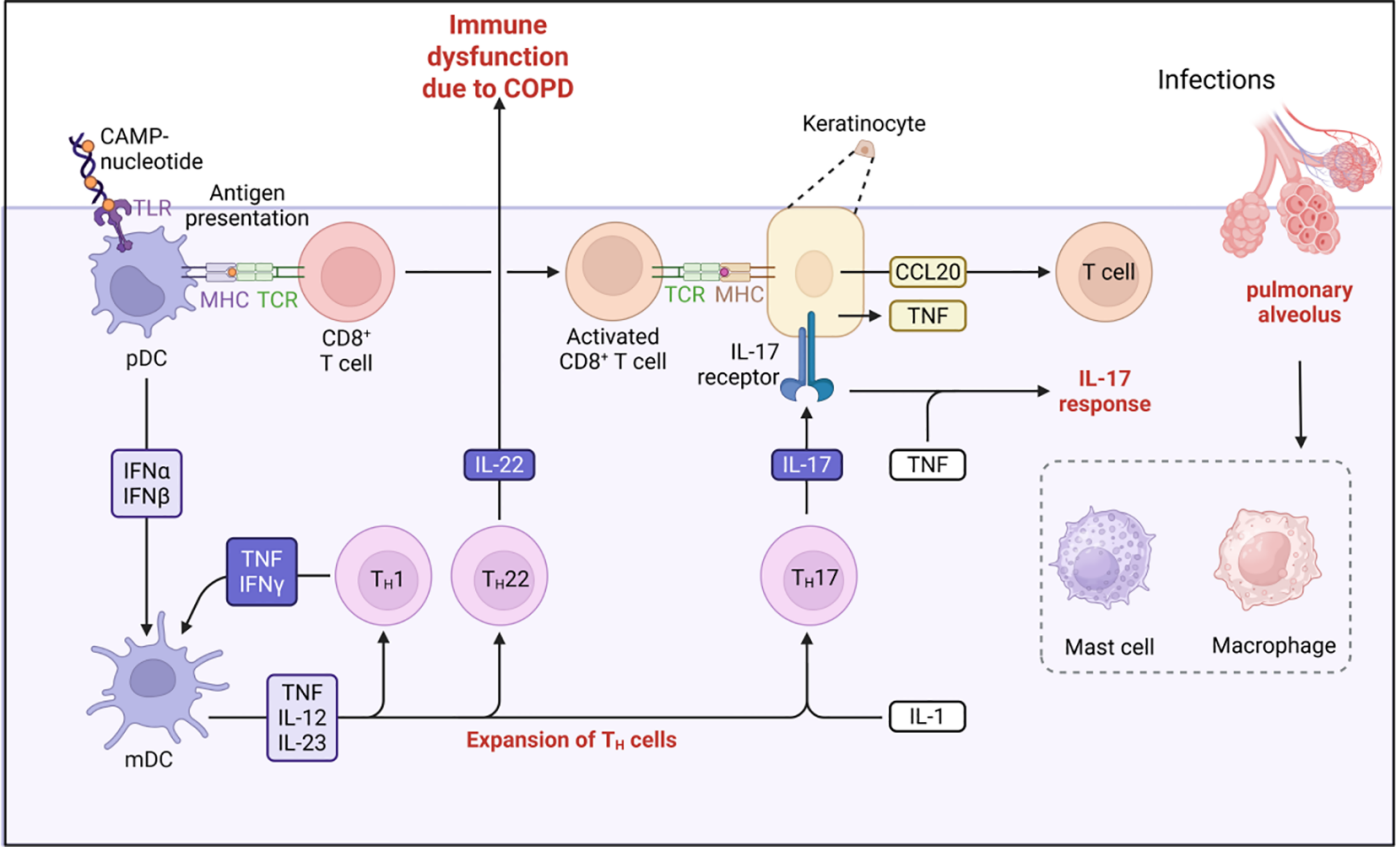
The immune response in relation to chronic obstructive pulmonary disease.

Respiratory epithelial cells play a dual barrier function in the pathogenesis of COPD, and the disruption of their structural integrity is a central component of disease progression. Long-term exposure to cigarette smoke and other irritants directly damages epithelial cells, triggering a three-part cascade of reactions: increased susceptibility to pathogens, persistent worsening of chronic inflammation, and reduced tissue repair capacity, all of which collectively drive the pathological progression of COPD. Epithelial cells dynamically regulate the immune microenvironment by releasing mediators such as TSLP (149). In this process, activated immune cells (such as mast cells) release substances like tryptase, which specifically degrade epithelial junction proteins (e.g., E-cadherin, zonula occludens-1). This degradation establishes a vicious cycle of “epithelial damage–immune activation” (150). Simultaneously, epithelial signals may activate the Th2/IL-13 pathway, promoting excessive mucus secretion and airway remodeling (**Figure 5**). Targeted therapy focuses on two main directions: at the molecular level, blocking TSLP signaling or inhibiting mast cell protease activity aims to reduce inflammation and restore the epithelial barrier; at the environmental level, reducing exposure to pollutants from the source (151). Existing research on the mechanisms of respiratory diseases provides important insights for COPD pathogenesis. Future studies should directly validate the specific role of the epithelial-immune axis in COPD to explore new therapeutic targets.

Epithelial-immune interactions and barrier dysfunction play a central role in COPD. Prolonged exposure to noxious gases (such as cigarette smoke) and particulate matter induces epithelial cell damage, triggering subsequent barrier dysfunction. Epithelial cells regulate local immune responses by releasing cytokines (e.g., TSLP) and chemokines. T cells and mast cells are pivotal in mediating epithelial barrier disruption and inflammatory responses. Mast cells release proteases (such as tryptase and chymotrypsin) that disrupt intercellular junctions of epithelial cells (including E-cadherin and zonula occludens-1), compromising barrier function. During acute exacerbations, mast cell activation exacerbates inflammatory responses and epithelial damage (152). Therapeutic strategies targeting epithelial-immune interactions and barrier dysfunction hold significant potential for COPD management. For example, agents targeting TSLP or inhibiting mast cell protease activity may reduce inflammation and restore barrier function (153). Additionally, mitigating environmental factors (e.g., reducing airborne pollutant exposure) and enhancing epithelial barrier function represent promising directions for COPD prevention and treatment.

### 2.6 Complement system

The complement system is a biochemical cascade that attacks the surface of foreign cells. It contains more than 20 different proteins and is named for its ability to “complement” antibodies in killing pathogens. Complement is a major humoral component of the innate immune response. In humans, the response is activated by binding of complement to antibodies already attached to these microorganisms or by binding of complement proteins to carbohydrates on the microbial surface. This recognition signal triggers a rapid killing response. The speed of the response is the result of signal amplification, which occurs after the activation of sequential protein hydrolysis of complement molecules (also proteases) (4). After the complement proteins initially bind to the microbe, they activate its protease activity, which in turn activates other complement proteases, and so on. This produces a catalytic cascade that is controlled by positive feedback. The cascade leads to the production of peptides that attract immune cells, increase vascular permeability, and condition (cover) the surface of the pathogen so that it is destroyed. This deposition of complement can also kill cells directly by disrupting the cytoplasmic membrane (154). In chronic lung disease, actin-related proteins are more commonly studied, and a study of AM phagocytosis in COPD mice demonstrated that PM2.5 exacerbates AM phagocytosis in COPD mice by inhibiting the Arp2/3 complex and F-actin, and exacerbating AM phagocytosis in COPD mice by the (Arp) 2/3 complex (4).

### 2.7 Surface barrier particulate matter

COPD is the most common chronic respiratory disease, a respiratory disorder characterized by permanent and progressive loss of lung function associated with smoking and exposure to harmful stimuli. Long-term inhalation of harmful gases and particulate matter are the main etiologic factors in the development and progression of COPD (155), and they are capable of inducing inflammation in the large and small airways and spreading it to the lung parenchyma, causing destruction of the alveolar walls, which in turn leads to emphysema. Studies have shown that a large number of inflammatory mediators are involved in inducing the infiltration of immune inflammatory cells and the production and release of their destructive enzymes, and that these inflammatory mediators are associated with the progressive destruction of the lungs in patients with COPD and trigger the remodeling of the central airways, distal airways, and lung parenchyma. In addition, oxidative stress from inhalation of noxious gases and particulate matter triggers a complex inflammatory response in the airways and lung tissues, ultimately leading to pathological changes in COPD.

## 3 The adaptive immune system and COPD: humoral and cellular immune responses

The adaptive immune system evolved in early vertebrates and allows for a stronger immune response as well as immune memory, in which each pathogen is “remembered” by a characteristic antigen. The adaptive immune response is antigen-specific and requires the recognition of specific “non-self” antigens in a process called antigen presentation. Antigen specificity produces a response against a specific pathogen or pathogen-infected cell. The ability to carry out these customized responses is maintained in the body by “memory cells”. If a pathogen infects the body more than once, these specific memory cells are used to quickly eliminate it. Adaptive immunity consists of antigen recognition, antigen presentation to T-lymphocytes, cell-mediated immunity, humoral immune responses, and immune memory. Among the most significant correlations between COPD and adaptive immunity are lymphocytes, helper T cells, and other.

### 3.1 Lymphocyte and antigen recognition

The cells of the adaptive immune system are specialized types of white blood cells called lymphocytes. B cells and T cells are the major types of lymphocytes and are derived from hematopoietic stem cells in the bone marrow. B cells are involved in humoral immune responses while T cells are involved in cell-mediated immune responses. Killer T cells recognize only antigens coupled to class I MHC molecules, whereas helper and regulatory T cells recognize only antigens coupled to class II MHC molecules (156). These two mechanisms of antigen presentation reflect the different roles of the two types of T cells. In contrast, B-cell antigen-specific receptors are antibody molecules on the surface of B cells that recognize whole pathogens without any antigen processing. Each B-cell lineage expresses a different antibody, so the complete B-cell antigen receptor represents all the antibodies that the body can make. Both B cells and T cells carry receptor molecules that recognize specific targets (157).

T cells can recognize a pathogen only after the antigen (a small fragment of the pathogen) has been processed and bound to the “self” receptor called a major histocompatibility complex (MHC) molecule. T cells can only recognize a pathogen after the antigen has been processed and bound to the “self” receptor called a major histocompatibility complex (MHC) molecule (26). T cells recognize “non-self” targets, such as pathogens, only after the antigen (small fragments of the pathogen) has been processed and bound to “self” receptors called major histocompatibility complex (MHC) molecules T and B lymphocytes provide specific immune protection by recognizing and eliminating pathogens and other harmful substances. These two types of adaptive immune cells are critical to the immune response in COPD (158).

### 3.2 Th immune response

**Figure 6:**
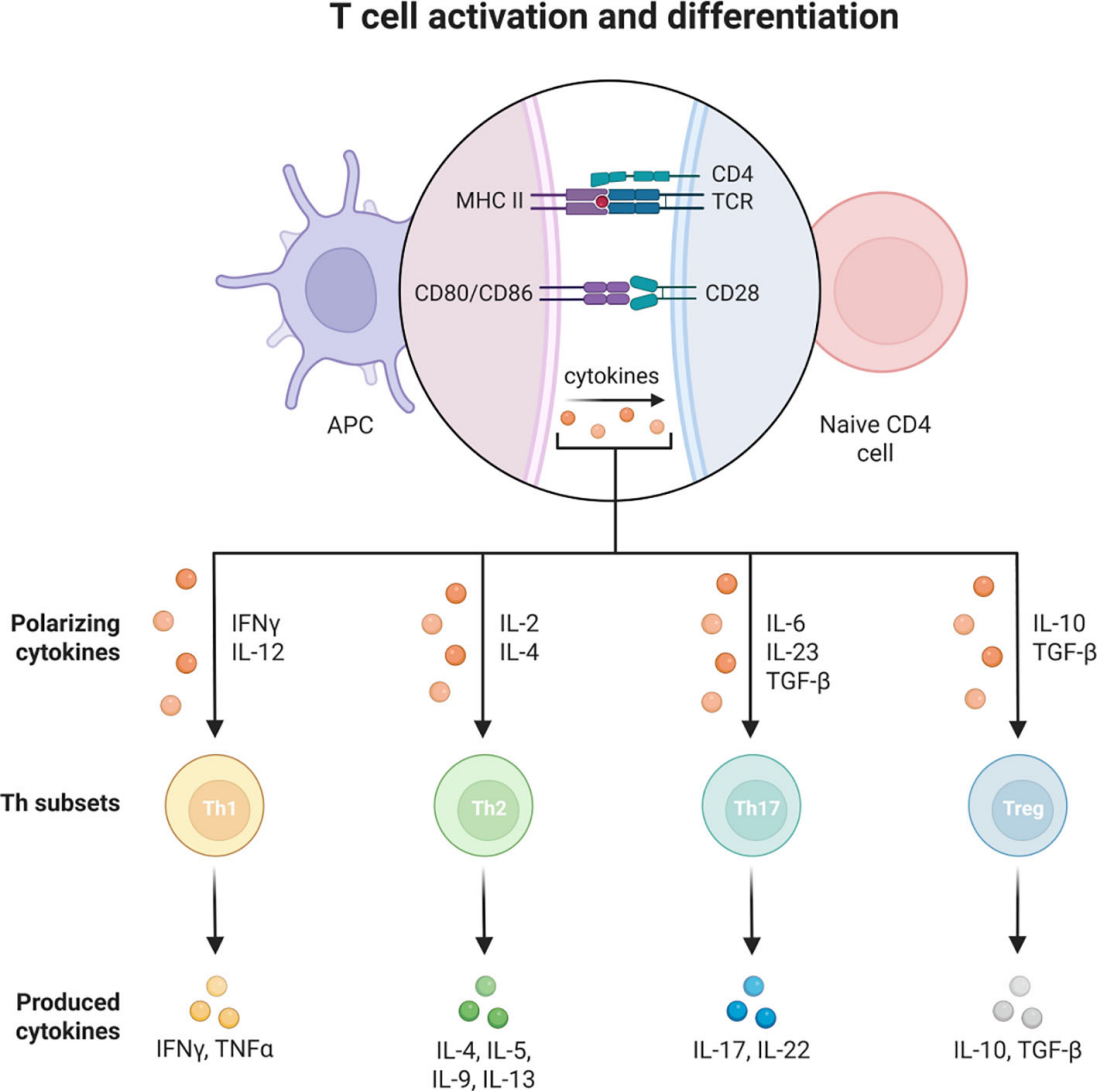
The role of T cells in COPD.

T cells are an important component of the adaptive immune system and can be divided into CD4 helper T cells and CD8 helper T cells. They are involved in immunomodulation and inflammatory regulation in COPD.CD4 helper T cells can differentiate into different subpopulations such as Th1, Th2, Th17 and regulatory T cells (Treg cells). In COPD, the increased activity of Th1 and Th17 cells leads to the production of cytokines, such as interferon-gamma (IFN-γ), by Th1 cells, which stimulate inflammatory responses and cellular damage, as detailed in the **Figure 6** (159).

CD8+ T cells play a key role in the inflammatory response and tissue damage associated with COPD. In the lung tissue of patients with mild to moderate COPD, there is a significant increase in CD8+ T cell subsets, particularly KLRG1+TIGIT+CX3CR1+ TEMRA (effector memory CD45RA+ T cells) and DNAM-1+CCR5+ TRM cells. These cells interact with myeloid cells and alveolar type II cells by secreting interferon-γ (IFN-γ) and exhibit highly expanded T cell receptor clonality (134). Additionally, CD8+ TEMRA cells play an important role in early-stage inflammation of the disease (160). In patients with severe emphysema, the number of CD8+ T cells is significantly increased and positively correlated with the degree of airway obstruction and alveolar wall damage (160). Interestingly, smoking and HIV-1 infection have also been found to promote the retention of CD8+ T cells in the airway mucosa, further exacerbating inflammation and tissue remodeling (161).

It is worth noting that T cells playing a crucial role in the chronic inflammation of COPD also include memory T cells (TRM) that persistently reside in tissues. COPD patients exhibit a significant increase in CD8+ TRM cells in lung tissue, which participate in local immune responses through the expression of CCR5 and DNAM-1 (156). In systemic sclerosis-associated interstitial lung disease (SSc-ILD), an increase in CD8+ and TRM cells has also been observed, suggesting their widespread role in chronic lung diseases (162). Interestingly, the function of TRM cells in COPD is not limited to immune surveillance but may also drive inflammation and tissue damage through interactions with other immune cells, such as NK cells and macrophages (162).

In COPD, there are complex interactions between cytotoxic CD8+ T cells and TRM cells. The high-frequency clonal expansion of CD8+ TEMRA cells and the local residency of TRM cells jointly promote the persistence of chronic inflammation (156). Additionally, the differentiation of CD8+ T cells into memory cells depends on the assistance of CD4+ T cells. This process may be further regulated in COPD patients. CD8+ TEMRA cells drive early inflammation by releasing cytotoxic mediators, while TRM cells maintain a crucial role in local immune surveillance and chronic inflammation.

Antigen presenting cells (APC) present antigens on their class II MHC molecules (MHC2). Helper T cells recognize these with the help of their CD4 co-receptor (CD4 +) expression. Activation of resting helper T cells causes them to release cytokines and other stimulatory signals that stimulate the activity of macrophages, killer T cells, and B cells (the latter of which produce antibodies.) (163). In COPD patients, the number of DCs in alveoli and distal airways is significantly increased, especially in the late stages of the disease. These DCs not only express mature markers (such as MHC II and B7) but also exhibit characteristics of both innate and adaptive immune phenotypes, indicating their important role in the immunopathology of COPD. Additionally, alveolar macrophages in the early stages of COPD exhibit impaired antigen-presenting function, which may lead to defects in immune activation and further exacerbate disease progression (136).

### 3.3 Cytokines

In the pathophysiological network of COPD, IL-17 (produced by Th17/γδ T/NKT cells) drives neutrophil recruitment and activation by inducing epithelial secretion of CXCL8 and IL-6, thereby exacerbating pulmonary inflammation and emphysema formation. IL-17 expression levels in bronchial mucosa are positively correlated with disease severity; whereas this factor enhances antimicrobial defense during acute infections, its dysregulation in COPD patients contributes to immune deficiency and increased infection risk. IL-17’s positive correlation with FEV1 suggests a potential role in lung function regulation (mechanism unknown) (164). In stable COPD patients, serum and sputum IL-17 levels are significantly higher than in healthy controls and rise further during acute exacerbations (AECOPD), indicating that IL-17 is not only associated with persistent inflammation in COPD but may also serve as a biomarker for disease progression (165). Additionally, IL-17 upregulation is closely associated with neutrophil-mediated inflammatory responses, providing a theoretical basis for IL-17-targeted inhibitor development.

IL-6 is a key inflammatory mediator in COPD, operating through the IL-6 receptor (IL-6R)-mediated signaling pathway. Studies demonstrate significant activation of the IL-6 trans-signaling pathway (IL-6TS) in COPD inflammation, particularly in neutrophil-dominant subtypes. IL-6TS activation correlates closely with neutrophil extracellular trap (NET) formation and bacterial infections (e.g., Haemophilus influenzae) (166). Moreover, high IL-6TS activity links to persistent neutrophilic inflammation, reduced quality of life, and elevated inflammatory mediators (e.g., IL-8, MMPs) in COPD.

IL-22 (from Th22/ILC3) maintains barrier function by promoting epithelial repair; its deficiency impairs tissue repair and delays bacterial clearance. Although inducing antimicrobial proteins to enhance defense, IL-22 suppression in COPD compromises immune responses and increases acute exacerbation risk (97). Critically, IL-22 and IL-17 form a synergistic axis: IL-22 deficiency amplifies IL-17-driven inflammation, whereas IL-22 supplementation restores bacterial clearance and attenuates inflammation (167)―This axis is vital during acute exacerbations, where bacterial infections (e.g., Streptococcus pneumoniae) cause delayed clearance due to IL-17/IL-22 defects, while viral infections (e.g., influenza) suppress their secretion by inhibiting IL-1β/IL-23, raising secondary infection risks (168).

Among other cytokines, IL-33 is markedly elevated in COPD patients with lung cancer, showing negative correlation with FEV1/FVC and increasing with GOLD staging (150), Conversely, IL-6/IL-1β rise in tuberculosis patients, positively correlating with total lung capacity (TLC) and RV/TLC, respectively—collectively serving as disease severity indicators.

### 3.4 Biomarker

Regarding biomarkers, low plasma levels of CPa9-HNE (a calmodulin fragment) indicate mild COPD with air trapping and elevated MMP-2; elevated suPAR correlates with GOLD staging (stage IV > stage I) and serves as a progression marker (169);dysregulated extracellular miRNAs (e.g., miR-374b-5p/miR-223-3p) during acute exacerbations aid staging differentiation (124). For treatment response prediction, patients with high eosinophil counts exhibit lower mortality and shorter hospitalizations. Endocan elevation during acute exacerbations shows negative correlation with FEV_1_/FVC, establishing it as an independent predictive factor (170), High NLR (neutrophil-to-lymphocyte ratio) predicts mechanical ventilation need and poor prognosis. Altered ABA (acid desquamation) levels—decreased in early-stage COPD but elevated in advanced disease—may indicate disease stage (171).

Studies on COPD biomarker-treatment response correlations reveal that Endocan, a marker of endothelial dysfunction, shows significantly higher levels during acute exacerbations versus stable periods and exhibits negative correlation with lung function (e.g., FEV_1_/FVC), establishing it as an independent acute exacerbation predictor; Abscisic acid (ABA) displays unique dynamics in COPD patients—reduced overall yet positively correlated with immune regulators, but elevated in advanced-stage disease, suggesting potential as a staging biomarker; The neutrophil-to-lymphocyte ratio (NLR) rises significantly during acute exacerbations, correlating directly with disease severity and poor outcomes. Elevated NLR further predicts mechanical ventilation need, establishing it as a key outcome assessment indicator for acute exacerbations.

### 3.5 BAL and sputum immunological profile

Bronchoalveolar lavage fluid (BAL) provides important insights into airway inflammation and immune responses in COPD. Immunological profiling of BAL and sputum delivers crucial information on COPD pathophysiology, disease classification, and therapeutic targets. Studies demonstrate abnormal expression of immune/inflammatory markers in COPD BAL. Extracellular vesicle (EV) protein and miRNA profiles in COPD BAL show significant alterations, particularly increased expression of inflammation-associated proteins (e.g., neutrophil degranulation markers) (172),Notably, BAL from COPD patients induces stronger neutrophil chemotaxis, potentially linked to disease severity. Moreover, secretory immunoglobulin A (SIgA) concentration is markedly reduced in COPD BAL and positively correlates with FEV_1_ (173). Protease-antiprotease imbalance contributes to COPD pathogenesis, evidenced by elevated matrix metalloproteinases (MMP-9, MMP-12) and tissue inhibitors (TIMP-1) in BAL (174).

Sputum analysis serves as a key tool for assessing airway inflammation and microbiome in COPD. Elevated mucin MUC5AC concentration in sputum (when measured with MUC5B) directly associates with disease severity, exacerbation frequency, and lung function decline; microbiome analysis reveals increased abundance of Proteobacteria and Haemophilus genera, closely linked to mucus plug formation and severity (175); inflammatory marker testing shows upregulated MMP-9, LTB4R, and A1AR mRNA, while CC16 mRNA is significantly reduced—the latter particularly associating with impaired lung function and eosinophilic inflammation.

Clinically, BAL and sputum profiling demonstrates utility in COPD management: First, MUC5AC, CC16, and EV-related proteins serve as diagnostic/prognostic biomarkers; second, sputum microbiome and inflammatory markers (e.g., eosinophil levels) guide personalized therapies (e.g., targeted biologics); lastly, longitudinal monitoring enables precise assessment of disease progression and treatment response. These approaches establish a clinical management loop from diagnosis to intervention and outcome evaluation.

## 4 COPD management strategies—drug treatment strategies

### 4.1 Innate immunity and the treatment of COPD

Chronic Obstructive Pulmonary Disease (COPD) is a common respiratory disease characterized by persistent airflow limitation, which has a significant impact on patients’ quality of life. As the disease progresses, patients experience symptoms such as dyspnea and decreased activity tolerance, which can even be life-threatening in severe cases. Scientific and rational drug therapy is essential to improve patients’ symptoms, enhance quality of life, and reduce acute exacerbation events.

#### 4.1.1 Bronchodilators

Bronchodilators are the core drugs used in the treatment of COPD to dilate narrowed airways by relaxing bronchial smooth muscle, relieve dyspnea, and improve the patient’s respiratory function. This class of drugs includes three groups: beta2-agonists, anticholinergics, and methylxanthines. beta2-agonists Beta2-agonists, such as salbutamol, formoterol, and salbutamol, are fast-acting bronchodilators that rapidly relieve shortness of breath and wheezing symptoms in COPD patients (176). These drugs increase the concentration of cyclic adenosine monophosphate in the lung smooth muscle by activating β2- receptors in the lungs, leading to the diastole of bronchial smooth muscle. They are suitable for the rapid relief of acute exacerbations of COPD as well as for long-term maintenance therapy, and can help to improve the lung function and quality of life of patients. However, long-term overdose of β2-agonists may lead to side effects such as palpitations, tremors or hypokalemia, and need to be used properly under medical supervision (177).

#### 4.1.2 Anticholinergics

Anticholinergics, such as tiotropium bromide and ipratropium bromide, reduce airway narrowing by blocking the action of parasympathetic nerves. These drugs help COPD patients reduce symptoms and improve quality of life by steadily dilating the airways over time and reducing sputum production (178). They are particularly beneficial in reducing the frequency of acute exacerbations and improving respiratory symptoms in COPD patients, but regular monitoring is needed to avoid potential side effects such as dry mouth and urinary retention. Methylxanthines Methylxanthines, such as theophylline, are less commonly used in current COPD treatment but still have value in some patients. These drugs can increase cAMP levels by inhibiting phosphodiesterase, which induces relaxation of bronchial smooth muscle and helps relieve airway narrowing (179). Theophylline also has a mild anti-inflammatory effect. Because of the narrow window between its efficacy and side effects, blood levels need to be closely monitored to ensure safe and effective treatment (180).

#### 4.1.3 ICS

Glucocorticoid selection Glucocorticoids are primarily used to control airway inflammation in COPD. Inhaled glucocorticoids (ICS) are effective in reducing airway inflammation, improving respiratory function, and reducing the risk of acute exacerbations. The use of ICS is of critical importance, especially in patients who experience frequent acute exacerbations or COPD combined with asthma. Although ICS (181) is extremely effective in controlling COPD, patients should not use it arbitrarily and must follow strict physician’s instructions. Inappropriate use (e.g., too high a dose or too long a duration of use) may result in a range of side effects, such as oral candida infections, hoarseness, osteoporosis, and an increased risk of cataracts. Clinicians will adjust the dose of ICS based on the patient’s symptom severity, history of acute exacerbations, and other co-morbidities to ensure that treatment is both effective and safe (182). Patients with COPD receiving ICS should be evaluated regularly for response to therapy, such as monitoring improvement in symptoms, changes in lung function, and frequency of acute exacerbations, to help the physician determine if adjustments to the treatment plan are needed, including changes in ICS dosage or consideration of the addition of other therapies, such as a long-acting bronchodilator or a phosphodiesterase-4 (PDE4) inhibitor, for further symptom control and improvement in quality of life (183).

### 4.2 Adaptive Immunity and COPD treatment

#### 4.2.1 Immunosuppressant

In the treatment of COPD, immunomodulatory strategies that focus on regulating T-cell and B-cell activity are expected to reduce the inflammatory response and oxidative stress in COPD, thereby alleviating symptoms and slowing disease progression. Immunosuppressive agents, including those that inhibit T-cell activity, may help reduce inflammatory lesions caused by over-activation of the immune response. Modulation of Th1/Th2/Th17/Treg balance is expected to reduce inflammation in COPD (184). Antibody drugs may also selectively interfere with B-cell activity, reducing antibody production and the release of inflammatory mediators. Overall, T cells and B cells play an important role in the pathogenesis and inflammatory process of COPD.123 Therefore, immunomodulatory and antioxidant therapies may be critical in the treatment of COPD. Balancing the immune response, suppressing hyperactivation of inflammation, and mitigating oxidative stress are expected to improve symptoms and quality of life in patients with COPD while slowing disease progression. This approach provides new ideas for personalized treatment in this field.

#### 4.2.2 IcnRNA

Dexmedetomidine, an alpha2-adrenoceptor agonist, is mainly used for sedation during perioperative procedures, endotracheal intubation and mechanical ventilation, and has a protective effect against inflammatory lung injury. It was shown that lncRNA PACER was highly expressed in the serum of COPD patients (185). Subsequently, it was found that overexpression of PACER in rat alveolar epithelial cells enhanced cell proliferation and migration through activation of protein phosphatase 2 by establishing a COPD rat model. After treatment with dexmedetomidine, it reduced PACER expression and proliferation and migration ability of alveolar epithelial cells in COPD rats, which could help COPD treatment. Andrographolide is a common anti-inflammatory agent that reduces the expression of inflammatory cytokines induced by cigarette smoke as well as infections. A study found that elevated inflammatory factors such as IL-6 and IL-8 in human bronchial epithelial cells exposed to CSE activated downstream signal transducer and activator of transcription 3 (STAT3) (186), which up-regulated the expression of lncRNA HOTAIR and enhancer of zeste homologue 2 (EZH2) (187). Andrographolide reversed CSE-induced HB inflammation and epithelial-mesenchymal transition by lowering IL-6 levels, and it prevented CSE-induced lung inflammation and small airway remodeling in an animal model, suggesting that andrographolide has potential clinical applications for cigarette-induced pulmonary dysfunction and COPD.

#### 4.2.3 Antimicrobial

Antimicrobial studies have found a strong association between acute exacerbations in COPD patients and bacterial infections (188), with common causative organisms including Catamoeba and Pneumococcus. Many drug studies are now beginning to consider the possibility of long-term application of low-dose macrolide antibiotics in the treatment of patients with chronic stable COPD. Studies have shown that this class of drugs is effective in anti-inflammatory and enhanced immunomodulation, which can greatly reduce the inflammatory mediators in the patient’s body and improve the patient’s exercise tolerance (189). However, even with the use of small doses of macrolide antibiotics, in the process of long-term medication, it is possible to develop drug resistance or cause an imbalance in the bacterial flora, and if the patient subsequently develops an acute attack, the use of antimicrobial drugs will be affected to a certain extent. For this reason, the use of this class of drugs in clinical treatment must be cautious. And at present, the time of clinical intervention is not yet specific, and its use time needs to be studied in depth.

### 4.3 Cytokine-targeted treatment strategies

**Figure 7:**
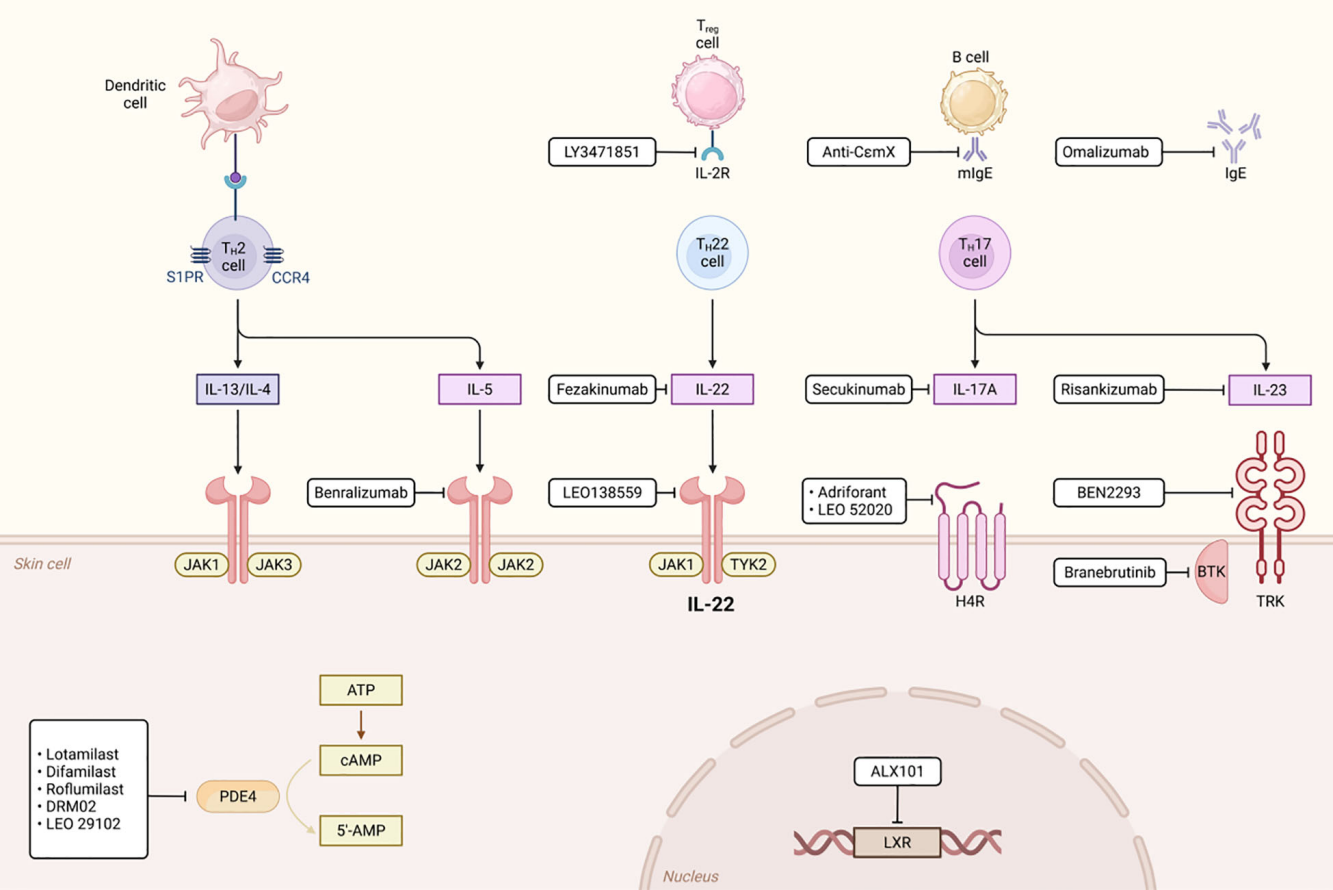
The Role of PDE4 in Immunity.

In mixed-type COPD, concurrent neutrophilic and eosinophilic inflammation requires treatment strategies targeting multiple inflammatory pathways. IL-1β is believed to play a key role in the inflammatory response of COPD (1). In recent years, IL-1β inhibitors such as canakinumab and MEDI8968 have become a hot topic in COPD treatment research. Canakinumab is a humanized anti-IL-1β monoclonal antibody that specifically neutralizes IL-1β signaling, thereby inhibiting the inflammatory response. Preliminary studies indicate that it can improve lung function and pathological damage in COPD patients by reducing pulmonary inflammatory cell infiltration and inflammatory cytokine release, and it also demonstrates anti-inflammatory potential in autoimmune diseases (190). However, there is currently a lack of large-scale clinical trial data to fully validate its efficacy and safety in COPD. MEDI8968 is a fully humanized monoclonal antibody that blocks the activation of IL-1α and IL-1β by binding to the IL-1 receptor 1 (IL-1R1). A Phase II randomized, double-blind, placebo-controlled trial indicated that while the drug did not significantly reduce the rate of acute exacerbations or improve quality of life in moderate-to-severe COPD patients, it demonstrated good tolerability with no serious adverse events, suggesting potential for further investigation (191) (**Figure 7**).

IL-17 and IL-6 inhibitors demonstrate therapeutic potential, offering new options for mixed-type patients. Specifically, IL-17 inhibitors may benefit neutrophil-dominant subtypes, whereas IL-6 inhibitors could target patients with activated IL-6TS pathways. Combination therapy may enhance anti-inflammatory effects, though further research is needed to evaluate synergy and safety (192). Blocking the IL-17 pathway effectively inhibits neutrophil-mediated inflammation, thereby alleviating chronic inflammation and acute exacerbations. Although preliminary studies support IL-17 inhibitors in COPD, larger clinical trials must confirm safety and efficacy (193). Suppressing the IL-6TS pathway may reduce neutrophilic inflammation and NET formation, potentially improving disease progression. For instance, tocilizumab (an IL-6R inhibitor) has proven effective in other inflammatory diseases, justifying further COPD investigation (166). However, current evidence remains exploratory, necessitating robust clinical validation. Future research should prioritize stratified treatment by inflammatory subtype to optimize individualized therapy.

Itepekimab is a human monoclonal antibody targeting IL-33. In a Phase 2 trial, it demonstrated potential in reducing exacerbations and improving lung function, particularly in former smokers with chronic obstructive pulmonary disease (COPD). For example, a Phase 2a trial showed that itepekimab reduced the annualized rate of exacerbations and improved FEV_1_ compared to placebo in former smokers. However, no significant benefits were observed in current smokers, highlighting the importance of patient stratification. An ongoing Phase 3 trial aims to confirm these findings in former smokers (2).

TSLP and IL-33 are key alarm molecules released by epithelial cells in response to environmental stimuli (such as allergens, cigarette smoke). They activate immune cells (such as dendritic cells, ILC2s) to promote type 2 (T2) and non-type 2 inflammatory responses, particularly in the eosinophilic COPD (eCOPD) subgroup, where TSLP and IL-33 expression are significantly elevated. ST2 is a functional receptor for IL-33, and its signaling pathway plays a crucial role in inflammation and fibrosis in COPD (25). Studies have shown that ST2+ILC2s are significantly increased in sputum from eCOPD patients and are positively correlated with elevated eosinophil percentages. Additionally, ST2 signaling is involved in modulating the production of T2 cytokines, such as IL-5 and IL-13, which play a key role in the pathophysiology of COPD (8). Therefore, blocking ST2 signaling may help inhibit T2 inflammation and fibrosis, thereby improving clinical symptoms in COPD patients.

Tezepelumab is a monoclonal antibody targeting TSLP, approved for the treatment of severe asthma and is being investigated in COPD. The TSLP and IL-33/ST2 pathways have partially overlapping yet independent regulatory roles in the inflammation and fibrosis of COPD. Studies have shown that simultaneously blocking TSLP and IL-33/ST2 signaling can more effectively suppress chronic T2 inflammation and fibrosis (51). In mouse models, combined blockade of TSLP, IL-25, and IL-33 significantly reduced pulmonary eosinophil infiltration and fibrosis. Therefore, a combined strategy of anti-ST2 and anti-TSLP blockade may provide a more comprehensive therapeutic effect for COPD patients (58).

Biological agents targeting interleukin-5 (IL-5), such as mepolizumab and benalizumab, have demonstrated significant clinical potential, but their application still faces numerous challenges. These drugs are primarily effective for COPD patients with eosinophilic inflammation, with efficacy concentrated in subgroups with higher baseline peripheral blood eosinophil counts (133). They only provide approximately 9%-12% improvement in reducing moderate-to-severe exacerbations, resulting in limited overall efficacy. Additionally, IL-5 inhibitors have minimal impact on lung function metrics such as FEV_1_, and some studies have even observed no improvement or a decline in lung function, suggesting they may struggle to reverse the core pathological change of airflow limitation. Another significant limitation lies in their single-target mechanism of action: the inflammatory network in COPD involves multiple cells and mediators, and blocking IL-5 alone may not fully suppress disease progression driven by non-eosinophilic inflammation (134). Furthermore, there is currently insufficient evidence regarding long-term safety and pharmacoeconomics, limiting their widespread application. Future research should focus on advancing precision medicine strategies, optimizing patient stratification based on biomarkers such as eosinophil counts to enhance treatment response rates. Concurrently, multi-target combination therapies should be explored, such as combining IL-5 inhibitors with anti-IL-4/IL-13 or anti-TSLP agents to synergistically regulate inflammatory pathways. Dosing regimens also require further optimization to balance efficacy and safety. Conducting long-term follow-up studies to assess sustained efficacy and adverse reactions, while identifying new biomarkers and targets, will be key to achieving personalized, efficient treatment.

### 4.4 NLRP3 inflammasome inhibitor

In recent years, the role of NLRP3 inflammasomes in the pathogenesis of COPD has been increasingly elucidated, emerging as a potential therapeutic target. NLRP3 inflammasome inhibitors, such as Dapantusin (MCC950), may offer new treatment options for COPD patients by inhibiting inflammasome activation. The NLRP3 inflammasome is a key component of the innate immune system, mediating inflammatory responses and pyroptosis. In COPD, excessive activation of the NLRP3 inflammasome triggers the release of inflammatory cytokines such as IL-1β and IL-18, exacerbating airway inflammation and oxidative stress (194). Studies have shown that NLRP3 expression is significantly elevated in the lung tissue and serum of COPD patients, confirming its key role in disease progression. MCC950, a highly selective NLRP3 inflammasome inhibitor, suppresses inflammasome assembly and activation. A study demonstrated that MCC950 significantly reduces the release of inflammatory cytokines by inhibiting NLRP3 inflammasome activation, thereby alleviating airway inflammation and oxidative stress (194), MCC950 also improves lung function in COPD model mice, reduces inflammatory cell infiltration, and mitigates airway wall damage. In an LPS-induced acute lung injury model, MCC950 significantly inhibited the accumulation of neutrophils and macrophages and reduced the levels of IL-1β and IL-18. These results suggest that MCC950 may provide a new therapeutic strategy for COPD patients by inhibiting NLRP3 inflammasome activation (128). Although MCC950 demonstrated promising effects in preclinical studies, its safety and efficacy in COPD patients require further validation. Evaluating the efficacy and safety of MCC950 in COPD patients, identifying biomarkers that can predict treatment response to MCC950, and exploring the combination of MCC950 with other anti-inflammatory drugs or antioxidants are priorities for future research.

### 4.5 scRNA-seq and CyTOF

Single-cell RNA sequencing (scRNA-seq) and mass cytometry (CyTOF) provide breakthrough tools for analyzing airway heterogeneity in COPD. scRNA-seq studies have revealed that airway basal cells in COPD patients exhibit a continuous molecular spectrum from health to disease, with stress genes (such as GADD45B/CITED2) significantly upregulated, potentially related to hypoxic adaptation; in the microenvironment of COPD combined with lung squamous cell carcinoma (LSCC), where tumor-associated macrophages (TAMs) and CD8+ T cell exhaustion markers are increased, while CD74+ tumor cells exhibit strong tumorigenicity and poor prognosis (195). High-dimensional protein analysis using CyTOF further revealed that in asthma-COPD overlap (ACO), AMs regulate the aging process via PPARγ; T cell subsets show Th1 cell imbalance, with inflammatory responses regulated by the NFIL3/Tim3 axis. The value of technological integration is highlighted in mechanism analysis: the combination of scRNA-seq and CyTOF in a mouse model confirmed that the HCK-mediated IL-17A/G-CSF/granulocyte axis is the core driver pathway of COPD inflammation and emphysema (175). Future efforts should focus on deepening target identification through single-cell multi-omics integration (transcriptome-proteome-epigenome), developing targeted therapies based on specific cell subpopulations (e.g., CD74+ tumor cells) and pathways (e.g., PPARγ), and ultimately achieving personalized clinical decision-making.

## 5 Discussion

Both innate and adaptive immune mechanisms underpin the chronic inflammatory characteristics of chronic obstructive pulmonary disease (COPD), and disruptions in these mechanisms play a key role in the pathogenesis of the disease. This complex immunopathological process involves coordinated interactions between macrophages, neutrophils, dendritic cells, and lymphocytes. Within the adaptive immune system, T lymphocytes (divided into CD4+ and CD8+ subpopulations) and B lymphocytes are the primary effector cells. Additionally, this process involves the participation of cytokines and signaling pathways. The redundancy of cytokine pathways constitutes the core bottleneck of monotherapy. Multiple factors (IL-17/TNF-α/IL-6/IL-8) amplify inflammatory responses through independent or overlapping signaling axes such as NF-κB, MAPK, and PI3K/AKT. For example, IL-17 mediates airway remodeling via the β-catenin/ACT1 pathway (175), while TNF-α drives tissue damage via NF-κB/MAPK. This complexity is further exacerbated by pathway interactions: NF-κB and MAPK interact at multiple nodes, and PI3K/AKT cross-talks with IL-17 signaling, leading to compensatory activation of other pathways when inhibiting a single pathway (e.g., using anti-IL-17 antibodies or NF-κB inhibitors), ultimately manifesting as effective animal models but limited clinical efficacy (196). Overcoming this challenge requires a multi-target strategy: combined targeting (such as dual inhibition of IL-17 and NF-κB) or using natural compounds (flavonoids/terpenoids) to simultaneously regulate the JAK3/STAT3/NF-κB and MAPK pathways. Future directions focus on three levels: elucidating pathway interaction networks (e.g., NF-κB-MAPK cross-nodal points); developing multi-target drugs based on network pharmacology-molecular docking; and customizing individualized treatment plans based on patient-specific pathway activity (e.g., IL-6/IL-17-dominant types).

IL-17 and IL-22 play a complex dual role in COPD, participating in both inflammatory responses and immune defense but are also closely associated with disease progression and acute exacerbations. During COPD progression, during bacterial and viral infections, IL-17 and IL-22 have distinct clinical significance. Therapeutic strategies targeting the IL-17/IL-22 pathway (such as IL-22 supplementation therapy) show promise in controlling acute exacerbations of COPD. Therefore, further exploration of therapeutic strategies targeting the IL-17/IL-22 pathway to improve clinical outcomes in COPD patients represents a promising future research direction. Cytokines and biomarkers in COPD play a significant role in assessing disease severity, predicting treatment response, and monitoring disease progression. For example, markers such as IL-33, suPAR, and miRNA can help distinguish between different stages and severities of the disease, while eosinophils and NLR can be used to predict treatment response and the risk of acute exacerbations. Future research should further validate the clinical utility of these biomarkers and explore their potential in personalized treatment. By integrating multiple biomarkers, it may be possible to develop more precise strategies for the diagnosis and treatment of COPD.

COPD is a highly heterogeneous disease, and its complex pathophysiological mechanisms and clinical manifestations pose significant challenges for clinical trial design. To improve trial efficiency and achieve personalized treatment, biomarkers and patient stratification strategies have become key tools. Among inflammation-related biomarkers, elevated levels of C-reactive protein (CRP) and fibrinogen are significantly associated with increased risk of acute exacerbations, making them useful for identifying high-risk patients and adjusting treatment; elevated white blood cell counts also indicate increased risk, particularly in patients with multiple coexisting inflammatory markers; and blood eosinophil counts are important biomarkers for predicting inhaled corticosteroids’ efficacy, with patients having higher levels potentially benefiting more significantly (197). Oxidative stress-related markers such as fibronectin 1 (FN1) are highly expressed in the serum and lung tissue of COPD patients and are associated with impaired lung function, potentially indicating disease progression (197); markers of imbalance between reactive oxygen species (ROS) and the antioxidant defense system can help assess disease progression and treatment response. Other prognostic markers include D-dimer (elevated levels associate with all-cause mortality) and red blood cell distribution width (RDW, associated with increased exacerbation risk, particularly useful for stratifying hypertensive patients) (132).

Patient stratification strategies should integrate clinical characteristics and biological mechanisms. Stratification based on clinical phenotypes (e.g., frequent exacerbation type, emphysema type) is limited due to phenotype overlap; whereas genotype-based stratification enables more precise targeted therapy. Combinations of multiple biomarkers (e.g., C-reactive protein, fibrinogen, and white blood cell count) significantly enhance stratification accuracy; specific proteins identified through plasma proteomics aid in early identification of rapidly progressive COPD. Functional tests such as the 6-minute walk distance (6MWD) are core stratification tools for predicting mortality, hospitalization rates, and acute exacerbation risk (198).

In trial design practice, biomarkers should optimize stratification aligned with individualized treatment goals in the latest GOLD guidelines (improving symptoms and reducing future risks). Targeted therapy trials should incorporate phenotype-specific biomarkers (e.g., for anti-inflammatory drugs) to precisely screen patients; eosinophil-directed ICS treatment strategies can be adjusted based on count levels to enhance efficacy while reducing side effects. Data-driven technologies such as machine learning models (SuStaIn) and network analysis can identify disease subtypes and progression trajectories, providing trial design support (175). Overall, the synergistic application of biomarkers and stratification strategies will drive precision development of COPD clinical trials. Future efforts should further validate biomarkers’ clinical utility and explore new technologies to advance precision medicine practices.

Clinical translation in COPD research faces multiple challenges, with the core issue being animal-human model mismatch. First, model design overlooks key clinical variables; despite COPD primarily affecting the elderly, studies predominantly use young animals (e.g., mice) (126). Gender factors are also neglected, with male animals dominating experiments, failing to reflect female patients’ unique pathophysiological mechanisms. Second, species differences exist in pathophysiological mechanisms. While cigarette smoke exposure models can simulate some human COPD features (e.g., inflammation, epithelial-mesenchymal transition), inflammatory biomarker expression (e.g., IL-6, IL-8) and regulatory mechanisms differ significantly between species, and human-specific molecular pathways remain poorly understood. Therapeutic translation bottlenecks are prominent: compounds effective in preclinical studies (e.g., andrographolide) often fail in human trials, as animal models cannot replicate patients’ individual differences and complex pathology (124). Technical limitations also apply. Diagnostic tools successful in animal studies (e.g., PET imaging for CCR2 assessment) have questionable human sensitivity/applicability. Differences in experimental parameters (e.g., drug dosage, exposure time) versus clinical practice further undermine result reliability. Gender differences in chemical reflex sensitivity during acute lung injury recovery are documented, but insufficiently explored in COPD models. Young animal models struggle to simulate elderly patients’ physiological decline and comorbidities. Optimizing model clinical relevance by incorporating elderly animals and balancing gender ratios; advancing technical clinical adaptability; and establishing humanized detection standards to bridge preclinical-clinical parameters represent critical future efforts.
